# Satellite-based indicator of zooplankton distribution for global monitoring

**DOI:** 10.1038/s41598-019-41212-2

**Published:** 2019-03-18

**Authors:** Jean-Noël Druon, Pierre Hélaouët, Grégory Beaugrand, Jean-Marc Fromentin, Andreas Palialexis, Nicolas Hoepffner

**Affiliations:** 1 European Commission – Joint Research Centre, Directorate D – Sustainable Resources, Unit D.02 Water and Marine Resources, Ispra, VA Italy; 2 Marine Biological Association of the United Kingdom, The Laboratory, Citadel Hill, Plymouth, United Kingdom; 3 CNRS, Laboratoire d’Océanologie et de Géosciences UMR LOG CNRS 8187, Université des Sciences et Technologies Lille 1 – BP 80, 62930 Wimereux, France; 4 MARBEC, Université de Montpellier, CNRS, IFREMER, IRD, Sète, France

## Abstract

This study investigates the association between an index of mesozooplankton biomass, derived from the Continuous Plankton Recorder survey and satellite-derived productivity fronts in the North Atlantic. While chlorophyll-a content (CHL) is commonly described as a proxy for phytoplankton biomass, the size of productivity fronts estimated from the horizontal gradient of CHL appears to be directly linked to mesozooplankton biomass. Our results suggest that the lifespan of productivity fronts, which ranges from weeks to months, meets the time requirement of mesozooplankton to develop. The proposed indicator describes the daily distribution of mesozooplankton’s suitable feeding habitat. It also provides a coherent interpretation of the productivity front development with respect to phytoplankton activity (CHL values) and potential predation by higher trophic levels. Since mesozooplankton are essential for feeding at higher trophic levels, this satellite-derived indicator delivers essential information for research and policy. An unanticipated positive trend of the indicator from 2003 to 2017 is observed at a basin scale under the current effects of climate change, with regional peaks in relatively poorly productive areas. Such monitoring indicator is potentially important to advances in marine food web modelling, fisheries science and the dynamic management of oceans towards sustainability.

## Introduction

The abundance and composition of plankton are strongly influenced by natural hydro-climatic variability^1–5^. Climate change alters the phenology of organisms, modifies the distribution of species and changes the composition of assemblages which, in turn, affects the quantity of energy available through the marine ecosystem and trophic interactions^6,7^. Chlorophyll-a fronts are meso-scale features such as ocean eddies or meandering currents that persist long enough (i.e. weeks to months) to potentially sustain zooplankton production^8–10^ prior to becoming hotspots for feeding at higher trophic levels. For instance, productivity fronts have been shown to attract large predators^11,12^ such as tuna (Atlantic bluefin^13,14^ and skipjack^15^) and marine mammals (fin whale^16,17^). The Continuous Plankton Recorder (CPR) survey was started in the 1930s to characterise the phenology and biogeography of zooplankton. Furthermore, both year-to-year and decadal changes in zooplankton abundance and composition were also examined as the time series expanded^1,2,4,18^. This large-scale survey made a notable contribution to investigating the relationship between zooplankton and its environment^5,19,20^, substantial ecosystem shifts^4,6,21^ and the effect of plankton changes on higher trophic levels^22–25^. However, the CPR data had never been interrogated on a large scale using an Earth observation variable to assess the dynamic links between mesozooplankton and productive oceanic features.

We investigate in this paper the association between an index of mesozooplankton biomass and the daily identification of productivity fronts from satellite-derived ocean colour. We use the horizontal gradient of chlorophyll-a (gradCHL) as the main proxy for food availability to characterise the environmental envelope of mesozooplankton. A specific range of suitable chlorophyll-a concentrations (CHL) is also used to exclude extremely oligotrophic and potentially eutrophicated areas. We combine large *in situ* (CPR) and environmental datasets at high spatial and temporal resolution (daily, few kilometres) to derive the habitat suitability index of mesozooplankton in the North Atlantic. The seasonal and decadal variability in habitat suitability as well as the spatial patterns identified are discussed, in the context of climate change and land-based eutrophication, with respect to their influence on the productivity of pelagic ecosystems and their relevance for policy support.

## Results

### Mesozooplankton biomass

**Figure 1:**
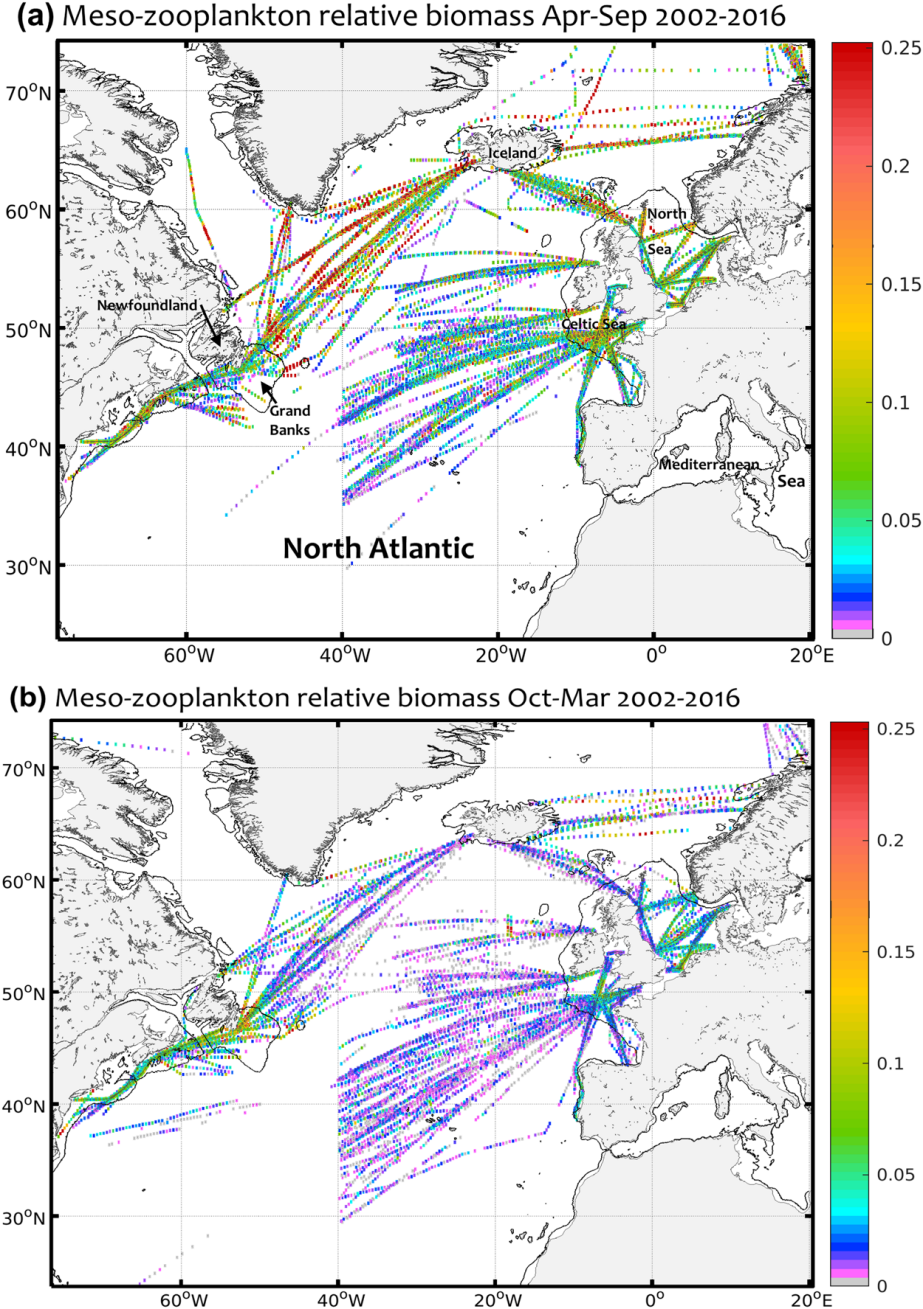
Mean relative biomass index of the combined small (generally below 2 mm) and large (generally above 2 mm) mesozooplankton observations (positive relative biomass only) collected between 2002 and 2016 by the Continuous Plankton Recorder survey in the North Atlantic (aggregation of 0.3° by 0.3°). **(a)** High season (April-September, upper map; n = 29,974). **(b)** Low season (October-March, lower map; n = 24,308). The maximum value of the colour bar was set to the 95th percentile of the high season (0.25).

The CPR dataset in the North Atlantic is composed of 54,282 samples of positive biomass from 2002 to 2016 covering both continental shelves and off-shore waters (Fig. SI 1). Despite its uneven spatial distribution along the main maritime routes, the large latitudinal range over all seasons of the dataset is ideal to develop a consistent habitat analysis of mesozooplankton (Fig. 1). A high-biomass season from April to September contrasts with a low-biomass season from October to March, except along the North-Western Atlantic shelf where high biomass levels are maintained (positive mean relative values only; Fig. 1). The highest relative biomass levels are found in spring and summer off the continental shelves in high latitudes, while the lowest levels occur in winter off the shelves in subtropical and high latitudes.

### Habitat parameterisation

**Figure 2:**
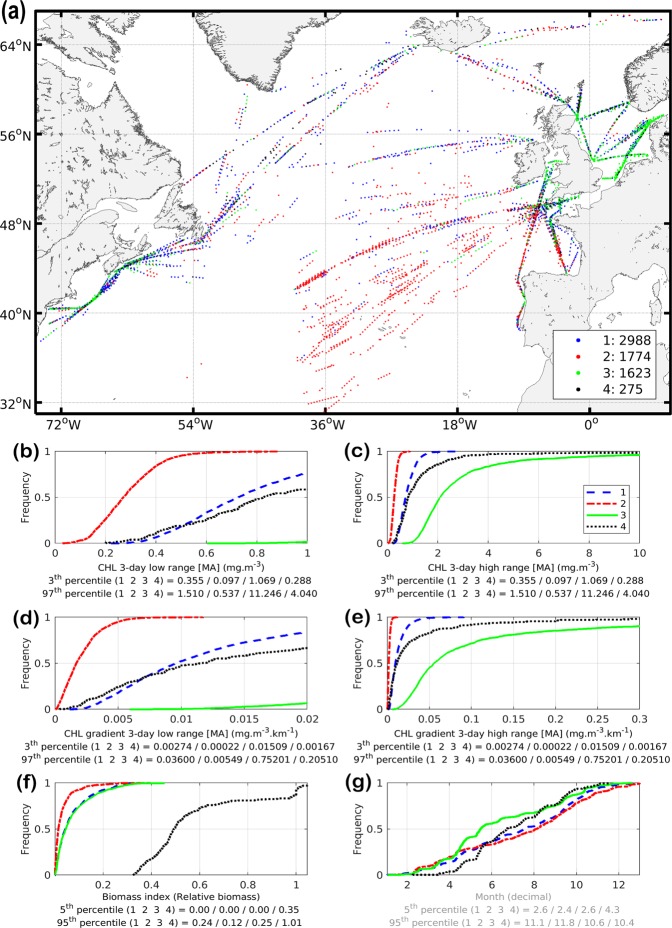
Cluster analysis. (**a**) Geographic distribution of clusters. (**b–g**) Cumulative distribution functions for each selected variables and cluster. (**b**,**c**) Chlorophyll-a concentration (CHL, low and high ranges, respectively). (**d**,**e**) Horizontal gradient of chlorophyll-a (gradCHL, low and high ranges, respectively). (**f**) The biomass index using night sampling and positive biomass only data (in relative values, *n*_observations_ = 6,660). (**g**) Sampling month (decimal month) as illustrative variable (legend in grey). The threshold values used for the model parameterisation are, for CHL, the 3rd and 97th percentiles of the extreme clusters (red and green clusters) and, for gradCHL, the 3rd percentile of the lowest cluster (red cluster). Note that 29 points in extreme latitudes were excluded from panel (**a**). See text and Fig. 3 for details.

**Figure 3:**
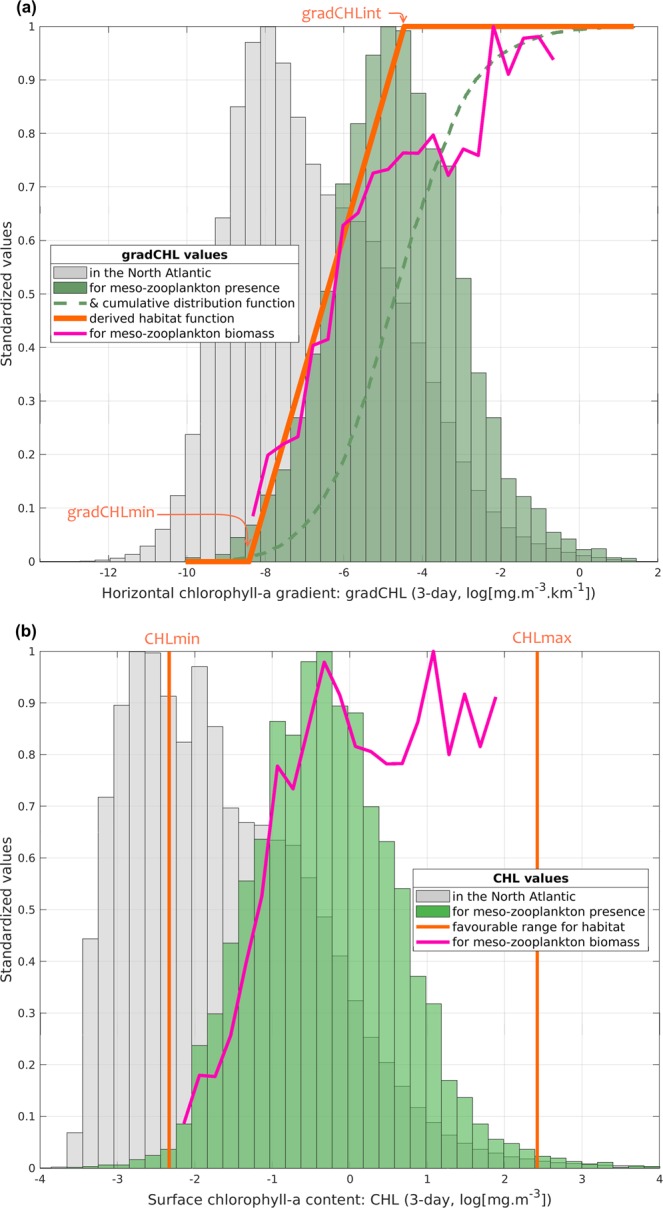
(**a**) Frequency in relative units of chlorophyll-a horizontal gradient (gradCHL; log-transformed) in the North Atlantic (grey histogram) and in locations where mesozooplankton are present (green histogram). The maximum slope of the cumulative distribution of mesozooplankton presence (green dashed line) was used to define the daily habitat linear function (orange line; see also Table 1). The mesozooplankton biomass (pink line) is superimposed. (**b**) Same as (**a**) for surface chlorophyll-a content (CHL; log-transformed).

The habitat model has four parameters: a suitable range of CHL (bounded by a minimum and a maximum value) and a minimum and intermediate value of gradCHL (Table 1). The cluster analysis is used to identify the first three parameters as boundary values of extreme environments (Fig. 2). Because CHL and gradCHL are linked (Fig. SI 5), we show for clarity their corresponding relationship in the overall North Atlantic together with the mesozooplankton biomass levels. The spreading of the clusters and model parameterisation is also overlaid on this graph to better display how the habitat index captures the environmental envelope. The maximum slope of the daily habitat is used to define the fourth parameter, the intermediate value of gradCHL (*gradCHL*_*int*_), which also corresponds to the peak presence of mesozooplankton (Fig. 3a). Cloud coverage and night samples has reduced to 6,660 observations the positive values of biomass index (from a dataset sampled at night of 34,779) which could be associated with a 3-day composite (±1 day of the satellite observation) of CHL and gradCHL. The cluster analysis (Fig. 2) showed that the suitable CHL range (the 3rd and 97th percentiles of the lowest and highest cluster, respectively) is between 0.097 and 11.246 mg m^–3^, while the minimum gradCHL value (the 3rd percentile of the lowest cluster) is 2.2.10^−4^ mg m^−3^ km^−1^ (Figs 3 and SI 5). This minimum gradCHL value and the maximum slope of cumulative distribution of mesozooplankton presence to define the lowest gradCHL value for which the daily habitat index reaches the maximum value of 1 (the intermediate gradCHL value, *gradCHL*_*int*_) at 1.15.10^−2^ mg m^−3^ km^−1^ (*ln*(gradCHL) of −4.47 in Fig. 3a).

**Table 1:** Habitat parameterisation used to define the environmental envelope of mesozooplankton in the North Atlantic Ocean, where CHL and gradCHL are the sea surface chlorophyll-a content and horizontal gradient from the MODIS-Aqua sensor, respectively.

Parameter values for mesozooplankton suitable habitat	Minimum value	Intermediate value	Maximum value
CHL (mg m^−3^)	0.097*	N/A	11.246*
gradCHL (mg m^−3^ km^−1^)	0.00022*	0.0115**	N/A

*See also Figs 2, 3 and SI 5.

**See also Figs 3 and SI 5.

The description of each cluster is as follows:

- The red cluster (27% of samples) corresponds to the lowest level of mesozooplankton biomass mostly located in the warm-temperate and subtropical Atlantic or subpolar latitudes in winter. The low CHL and gradCHL levels of this cluster indicates relatively small productivity fronts and low productivity levels. The 3rd to 97th percentile of gradCHL ranges from 0.00022 to 0.00549 mg m^−3^ km^−1^ (from −8.4 to −5.2 in logarithm form), which corresponds to low but increasing levels of mesozooplankton biomass index (Fig. 3a).
- The green cluster (24% of samples) represents part of the medium-high levels of biomass index mostly located on the shelves of temperate and subpolar latitudes, but also more scarcely in the open ocean. This cluster, which displays higher frequencies in spring (Fig. 2g), corresponds to the highest levels of CHL and gradCHL (i.e. relatively high productivity).
- The black cluster (4% of samples) is related to exceptionally high levels of biomass index in temperate and subpolar latitudes. The frequency increases of this cluster in spring are similar to those of the green cluster, albeit about 3–4 weeks later (Fig. 2g). This high-mesozooplankton biomass cluster corresponds to medium levels of CHL and gradCHL.
- The blue cluster (45% of samples) occurs in almost all latitudes, with a higher frequency in autumn (Fig. 2g). It has medium-high levels of biomass index similar to the green cluster but with medium levels of CHL and gradCHL (lower than the black cluster). The 3rd to 97th percentile of gradCHL ranges from 0.00274 to 0.036 mg m^−3^ km^−1^ (from −5.9 to −3.3 in logarithm form).

Both the CHL and gradCHL values are distributed over a much larger range when using all satellite observations for the whole North Atlantic Ocean than when using only areas for which mesozooplankton are present (Figs 3 and SI 5). The relationship between the index of mesozooplankton biomass (pink line in Fig. 3a) and gradCHL has three notable sections, starting with a positive slope from −8.4 to −5.8 (in log form), followed by a first (up to −2.5) and a second plateau (above −2.5). This positive linear section displays a close link between relatively small productivity fronts and low to medium mesozooplankton biomass levels. This connection disappears for medium-high levels of gradCHL (i.e. well-developed productivity fronts) when biomass plateaus. The maximum biomass level forms a second plateau which corresponds to the largest gradCHL values. The corresponding curve of mesozooplankton biomass as regards CHL distribution (Fig. 3b) is markedly more variable and noisy than that for gradCHL with (i) a maximum biomass level reached before a similar plateau occurs and (ii) substantial variation in biomass at high CHL levels. Table 1 summarises the habitat parameterisation.

### Outputs of the habitat index

**Figure 4:**
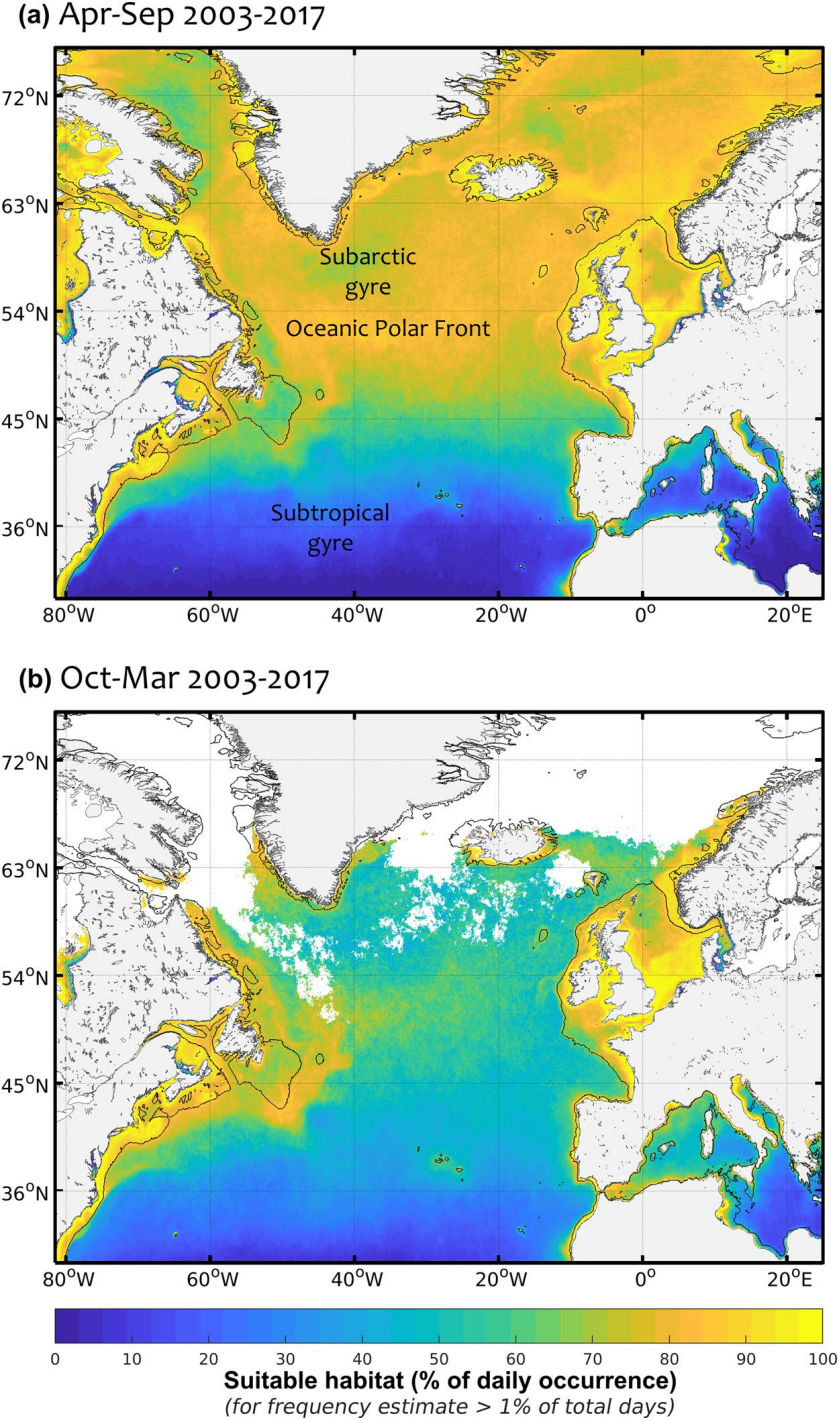
Seasonal suitable habitat index of mesozooplankton in the North Atlantic for (**a**) April–September and (**b**) October–March 2003–2017. The suitable habitat index is defined by the presence of large productivity fronts and is expressed as the frequency of occurrence weighted by the front size. Blank areas denote habitat coverage below 1% of the total number of days in the considered time period, except the Baltic Sea, which was not considered (see text).

The daily habitat index of mesozooplankton is defined as grid cells for which CHL is within the identified suitable range (above 0 and up to 1) and gradCHL is above a minimum threshold following the function highlighted in Fig. 3a and the equation defined in the methods section. The two main seasonal maps of suitable habitat are computed from this daily index averaged in time as an equivalent of a weighted mean. These time composites are therefore expressed in frequency of suitable habitat occurrence computed as a mean of 0-to-1 daily values which are associated with the front size (%, Fig. 4). The seasonal index of suitable habitat is at its maximum (frequency of suitable occurrence >75%) from April to September north of 45°N in the open ocean and at its minimum (45–75%) from October to March. Winter months are marked by maximum levels of habitat index (frequency of suitable occurrence of about 35–55%) south of 45°N and by a low coverage of CHL (<1% of days) north of 60–65°N with presumably relatively low plankton activity because of light limitation. The lowest habitat index levels occur south of 40°N during summer in the North Atlantic and the Mediterranean Sea with mean levels below 35%. Most continental shelves show sustained levels of suitable habitat index year round (frequency of suitable occurrence >90%). The notable exceptions are the northern-central North Sea, the outer shelf of the Celtic Sea and the Grand Banks of Newfoundland where the habitat index level is in the range 55–80%. Only part of the near shore areas in summer months show low suitable habitats related to CHL levels above *CHL*_*max*_ in relation to potential eutrophication. The results from the Baltic Sea have not been considered since corrections to the current approach should be applied to mitigate the potential effects of (i) the coloured dissolved organic matter that affects CHL estimates and (ii) cyanobacteria bloom that may affect zooplankton populations.

**Figure 5:**
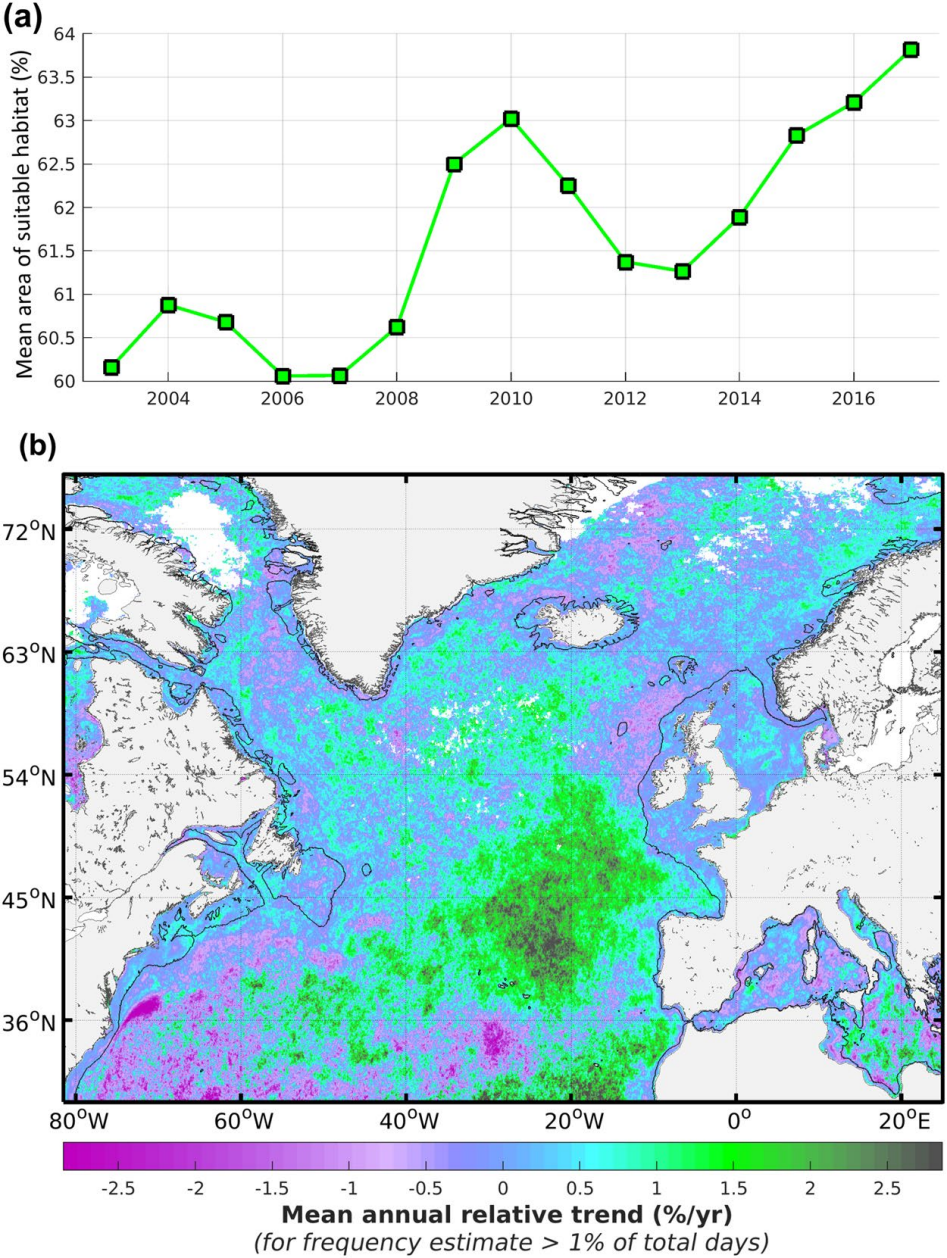
Annual variability of mesozooplankton feeding habitat index in the North Atlantic for the period 2003–2017. (**a**) Total annual fraction of the ocean surface characterised by a suitable mesozooplankton habitat. (**b**) Regional trends in relative value (% per year) computed from annual means. Positive regional trends represent an increase in frequency of occurrence of productivity fronts. Blank areas correspond to cloud or sea ice cover, or to habitat suitability with CHL detection below 1% of the total number of days in the considered period, except the Baltic Sea which was not considered (see text).

The surface suitable for mesozooplankton habitat in the North Atlantic shows a fluctuating annual level with an overall positive trend over the period 2003–2017 (Spearman’s r = 0.79, p < 0.001, Fig. 5a) that results from an increase in frequency and/or intensity of productivity fronts. This trend is however highly uneven in space over the North Atlantic. A clear decrease with levels below −1.5% per year characterises the oligotrophic south-west North Atlantic, while other more extended areas such as the temperate and tropical latitudes of the eastern basin show a strong increase with values above +2% per year (Fig. 5b). The positive trends of the mesozooplankton habitat index generally correspond to areas of medium-low habitat levels in the open ocean while the highest negative trends mostly occur in low habitat levels.

**Figure 6:**
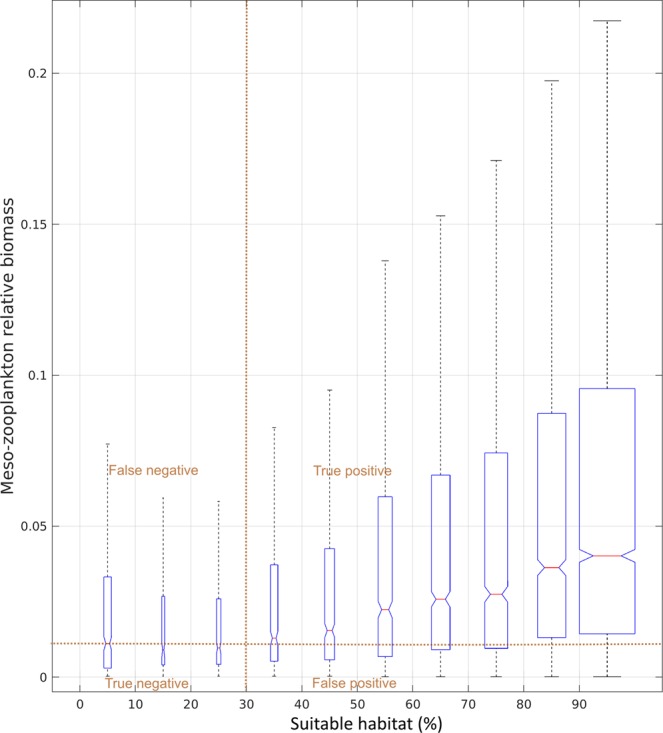
Distribution of matchups between the mesozooplankton relative biomass (only positive values sampled during night and day) from 2002 to 2016 and satellite-based habitat index (3-day habitat centred on the day of the observation; n = 16,146). Box width is proportional to the number of matchups. On each box, the central mark indicates the median and the bottom and top edges of the box indicate the 25th and 75th percentiles, respectively. Where the notches in the box plot do not overlap, it can be concluded with 95% confidence that the true medians differ. The whiskers extend to the most extreme data points not considered as outliers (1.5 times the interquartile range, i.e. 99.65% for a normal distribution).

The biomass index of mesozooplankton increases with the habitat suitability index (Fig. 6). The same stable pattern at around 70% of habitat index (around 0.7 with daily values) is observed for the median biomass index values of the boxplot in Fig. 6 and in Fig. 3a although we used 16,146 matchups (night and day data) instead of 6,660 (only night data). In order to provide a quantitative performance of the habitat model, we computed a simple statistical binary classification (an error matrix) separating in four spaces the true/false positives and negatives of the model. The maximum rate of correct matchups (sum of true positives and true negatives) of 75% is obtained for a central point delimitating these four spaces at a habitat index value of 30% and a biomass limit of 25th percentile (value of 0.011; see Fig. 6). False negatives originating in the model make up 3% while false positives make up 22%.

## Discussion

The results of this study detail the predictive capacity of the size of productivity fronts (horizontal gradient of CHL) to estimate the mesozooplankton feeding habitat. While this satellite-derived proxy of mesozooplankton activity provides an insight on the exploited fraction of primary production by marine ecosystems, this indicator has several limitations.

### Modelling methods

The limitations of our approach notably relate to the absence of detection in cases of cloud or ice coverage, in low light conditions (high latitudes) and when the bulk of primary production occurs deeper than the satellite sensor’s optical depth. In the latter case, both the satellite sensor and the CPR (sampling of the first 10 m^26^) do not account for the deep CHL maximum. Consequently, our approach underestimates the mesozooplankton biomass and the suitable habitat index in oligotrophic water such as in the warm-temperate and the subtropical areas. The downward trend of mesozooplankton-suitable habitat observed in the North Atlantic Subtropical gyre (partially shown on Fig. 5b) is however consistent with the negative trend of net primary production estimated from a carbon-based primary productivity model with vertically resolved photoacclimation^3^. This decreasing productivity in oligotrophic environments was shown to be caused by the deepening of the mixed layer depth and an increase in sea surface height anomaly and sea surface temperature^3^. Environments where productivity may mostly occur in the subsurface layer are generally considered to be substantially less productive than near the surface because of light limitation (exponential decrease of light with increasing depth). Areas where a deep CHL maximum occurs may thus characterise a relatively low source for mesozooplankton feeding, albeit locally important. Another potential limitation of the approach is the presence of suspended inorganic matter and coloured dissolved organic matter^27^ which may bias the CHL estimation in land-influenced marine waters. This bias mostly occurs in coastal areas near river plumes and in the larger Baltic Sea which was not considered for this reason. Finally, the spatial resolution of the satellite-derived CHL data, which is currently 1/24°, may also limit the sensitivity of the habitat index to relatively small productive features that may be particularly important in oligotrophic and coastal areas. The comparison between the surface area of four to seven satellite-derived cells of about 2.5 by 1.8 nautical miles (i.e. about 22 to 38 square nautical miles) and the 10 nautical miles transect of the CPR sampling has, to some extent, disrupted the relationship between gradCHL and the mesozooplankton biomass index. We also specifically excluded potentially eutrophicated waters with a maximum CHL value as the threshold, assuming that these waters mostly corresponded to disrupted food chains. In the near-shore areas however, the local dynamics is not appropriately described by the 10 nautical miles sampling and current satellite resolution. The selection of the satellite pixels closest to the 10 nautical miles track of the CPR remains the best possible comparison (see Fig. SI 3), but using higher resolution satellite data such as those from the European Sentinel 3 sensor (with up to 300 m resolution) will allow for a better analysis of this relationship. The frequency of observation from geostationary platforms (MeteoSat Third generation satellites in 2020) will offer the possibility to analyse the CHL front dynamics around the CPR sample at hourly time scale^28^.

Despite these limitations, the application of a single and simple model across various environments enables us to identify suitable mesozooplankton feeding environments in the North Atlantic Ocean. In this study, we benefit from a spatially and temporally wide dataset of biomass that covers a large geographical area and various geomorphological environments (shelf and open ocean). Moreover, the 15-year time-series of matchups between the CPR and MODIS-Aqua sensor data (2002–2016) across all seasons allows consistent model calibration and validation. The use of a clustering method for identifying relevant thresholds with abundance-only data ensures that the environmental characteristics that may be important for mesozooplankton, even when potentially under-represented in the observation data, are captured. This is an essential part of the procedure to accurately identify the overall environmental envelope. We assume that large productivity fronts are more resilient than smaller features and thus more capable of sustaining well-developed food chains on which mesozooplankton can feed^29^. This assumption, which is formulated in similar habitat analysis^13,15^, was confirmed by the overall relationship found between the slope of the 0-to-1 daily value of habitat index and the mean biomass index of mesozooplankton (Fig. 3a). Our results suggest that the linear function that links daily habitat quality with gradCHL levels provides an estimate of feeding capacity from productivity fronts of variable sizes expressed in terms of horizontal rate of change. The maximum rate of 75% of correct matchups (Fig. 6) shows a good level of performance, especially considering that part of the 22% of false positives of the model can be attributed to predation.

### Dynamics of planktonic food webs: the importance of scales

The link between productivity fronts and zooplankton highlights the critical importance of time and spatial scales inherent to meso-scale features for the flow of organic matter across planktonic food webs. Considering that the timescale for growth increases with trophic levels (days for phytoplankton, weeks for zooplankton) it is essential for organisms with size-limited mobility that productive features stand long enough to sustain their growth^8–10^, i.e. that the prey’s productivity covers the timescale of the predators’ growth. Highly mobile species such as fish can actively swim towards their prey, while planktonic predators can survive and grow only if prey are available nearby. Our results show that meso-scale productivity fronts are sufficiently resilient to ensure an efficient energy transfer from phytoplankton to mesozooplankton. This finding is consistent with previous studies that stress the attractiveness of productivity fronts for large predators, such as tuna species or fin whales^11,13–16^, which implies that these fronts support the development of complete pelagic food webs. The stabilised levels of mesozooplankton biomass for relatively high values of gradCHL (Fig. 3a, plateau of gradCHL from 0.0041 to 0.0821 mg m^−3^ km^−1^) may therefore result from predation by high trophic levels (HTL) exploiting these resilient productivity fronts. The corresponding range of gradCHL agrees with the identified niches of skipjack tuna in tropical waters^15^, of adult Atlantic bluefin tuna in the North Atlantic^13^ and of sardine and anchovy in the Mediterranean Sea (unpublished data). This study supports that sufficiently old productivity fronts are able to synchronously host well-developed phytoplankton and zooplankton populations and therefore represent potential feeding hotspots attracting HTLs. Our results (Fig. 2g) suggest that zooplankton development lags behind the establishment of frontal structures by about 3–4 weeks, which is close to what was observed during the North Atlantic Bloom Experiment^30^. Similar patterns of increased frequency, separated by an interval of 3–4 weeks from April to July between the clusters from high to medium CHL/gradCHL levels and from medium to high zooplankton biomass (green and black clusters respectively, Fig. 2g) are particularly striking. The green cluster is interpreted as corresponding to relatively young and developing mesozooplankton populations during phytoplankton bloom events, while the black cluster represents fully developed mesozooplankton populations that likely control phytoplankton growth in relatively mature fronts. The blue cluster could conceivably correspond to the oldest productivity features associated with fully developed food web where HTL predation occurs on mesozooplankton (see the first plateau of biomass index, Fig. 3a). The maximum biomass level of mesozooplankton (second plateau which corresponds to the largest gradCHL values in Fig. 3a) may result from the few cases where mesozooplankton productivity exceeds the HTL control capacity (corresponding to the highest distribution tail of the black cluster for biomass and gradCHL). How long a front has been established is difficult to assess because hydrological patterns and processes are intertwined. However, our results show that a degree of front ‘maturity’ may be estimated in that particular case because the CPR frequently sampled one of the most important productivity events (North Atlantic spring bloom) that extends over a large latitudinal range for several months. The sequence of productivity fronts among the clustering would therefore be from green to black and then from black to blue, the latter mostly marking the predation potential and advancement in the food chain. In terms of large scale features, the highest values of the index observed in the North Atlantic Ocean reflect remarkably well the presence of the Oceanic Polar Front^31^ and the boundary of the Subarctic gyre^32^ in both main seasons (Fig. 4).

The overall positive trend of productivity front occurrence in the North Atlantic from 2003 to 2017 (Spearman’s r = 0.79, p < 0.001) was unexpected given the observed surface warming of the oceans^33^ which is meant to increase the seasonal thermocline and obstruct the resurgence of deeper and nutrient-rich waters. We hypothesise that this overall increasing frequency of surface productivity fronts over time is atmospherically driven. More precisely, it could be linked to an increase of the latitudinal oscillation between low- and high-pressure systems generating unusual wind, evaporation and precipitation events. These phenomena are likely to increase vertical turbulence in the near-surface water column through shear stress or water resurgence. The latter may result from the observed intensification of the general thermohaline circulation and global water cycle^34,35^. This interpretation agrees with the opposite patterns of changes in surface salinity identified between the east and west of the North Atlantic from 1993 to 2015 and with both the salinity patterns and increased intensity of boundary currents and free jets observed in the central Mediterranean Sea over the period 1987–2014^33^. The amplitude of such basin-wide trend needs however to be taken with caution as the time-series is relatively short and radiometric corrections may not have fully compensated the satellite sensor degradation^36^.

### Perspectives for research and policy

The direct translation of Earth observation data into a useful metric as described in this paper is closer to an algorithm than a sophisticated numerical model. The development of an algorithm can be justified because our direct transformation of the remotely sensed sea surface observation using *in situ* observations of mesozooplankton is similar to the transformation of remote-sensing reflectance into a CHL estimate. Importantly, the main transformation is based on a gradient calculation rather than on absolute values of CHL, making this method less dependent on the uncertainty in CHL calibration across a wide spectrum of optical environments or phytoplankton pigment types. To date, these gradients derived from ocean colour data have been barely used in habitat studies although satellite observation is inherently powerful in feature identification.

We anticipate that this algorithm will be an efficient tool for monitoring the impact of climate change on marine living resources at both global and regional scales and therefore contributing to the conservation and sustainable use of the oceans, seas and marine resources (United Nations sustainable development goal 14). Coverage could be further extended with CPR data in other oceans such as the North Pacific, the Southern Ocean and the tropical Atlantic (e.g. Gulf of Guinea) noting that limited regional variation is likely to be found considering the large spectrum of environments already accounted for. Specific developments are needed in coastal areas using higher resolution CHL data and in the Baltic Sea because of optical (coloured dissolved organic matter) and planktonic (cyanobacteria) particularities. Further analysis of the trend is also needed using in parallel other satellite sensors’ data (SeaWiFS 1997–2010, VIIRS since 2012, Sentinel 3 OLCI since 2016).

Mesozooplankton can be considered as the keystone of marine food webs linking low and high trophic levels. By describing the dynamics of mesozooplankton distribution, this observation-based habitat indicator allows a relative but reliable estimate of the main flow of organic matter in pelagic ecosystems. Robust spatial and temporal information on zooplankton distribution are efficient forcing or validation data to reduce uncertainties in ecosystem or biogeochemical models. We therefore recommend that at least the basic metric (the horizontal gradient of daily chlorophyll-a content) be computed and made available to the scientific community by a marine consortium, such as the Copernicus Marine Environment Monitoring Service (http://marine.copernicus.eu/) as a basis for a plankton-to-fish indicator. This metric is not currently available and is challenging to compute as it requires advanced expertise in satellite ocean colour science.

Ambitious marine policies in terms of objectives or spatial coverage could greatly benefit from this type of satellite-based indicator. The European Commission’s Marine Strategy Framework Directive (MSFD)^37^ includes quality of habitats in line with prevailing environmental conditions and marine food webs at normal abundance. Criteria and methodological standards for achieving good environmental status (GES)^38^ by 2020 seek such indicators to support assessment and monitoring obligations. These indicators provide ecologically relevant, comparable and cost-effective information for characterising the status of the marine environment (pelagic habitats, the balance of total abundance between trophic guilds) and the potential impacts from cumulative pressures such as eutrophication and climate change. Moreover, the distribution of zooplankton could also support fisheries policies such as the EU common fisheries policy (CFP) by providing information on the environmentally driven potential productivity of fish. In response to the substantial climate-driven variability in potential productivity, such an indicator – integrated over a year, which is a typical time for fish recruitment – may provide an explanation for varying fish stocks^39^ and enhance dynamic fisheries management through the spatial adjustment of fishing efforts^40,41^. Such dynamic management may be an efficient method to limit over-fishing and favour fishermen acceptance. By linking low to high trophic levels, the use of this satellite-derived indicator may therefore support a more tangible CFP implementation of the ecosystem-based approach to fisheries management (Regulation (EU) No 1380/2013^42^).

## Materials and Methods

The environmental habitat analysis has four steps: (i) collect and process data (index of mesozooplankton biomass data, gridded chlorophyll-a concentration and chlorophyll-a horizontal gradient from satellite remote sensing); (ii) perform a cluster analysis to characterise the environmental envelope of mesozooplankton; (iii) formulate the habitat index equation to project the environmental envelope on a gridded map on a daily basis; and (iv) perform a seasonal and temporal analysis of habitat suitability index and compare with a larger set of biomass data than those used for model training.

### Step 1 – Data

#### Mesozooplankton biomass index

The CPR survey has been in routine operation for 60 years and the materials and procedures used have remained consistent during this time. It is accepted that CPR samples zooplankton in the integrated first 10 m of the water column over a transect of 10 nautical miles (see supplementary information for details). In total, 68,364 samples in the North Atlantic from July 2002 to December 2016 were extracted and 54,282 positive biomass samples (79.4%) were used (Fig. 1) to match the lifespan of the MODIS-Aqua ocean colour sensor. Two planktonic groups were created for the small (generally below 2 mm; 40 taxa) and large (generally above 2 mm; 91 taxa) mesozooplankton (Tables SI 1 and 2), representing the most present species in the North Atlantic (Fig. SI 2). The distinction between two size classes was justified by the two major divisions made in the CPR analysis of zooplankton. Small copepods were identified using a sub-sampling method termed “traverse” while the whole CPR silks (i.e. both filtering and covering silks) was used for larger copepods (see supplementary information). Copepods represented a substantial fraction of the zooplankton in the North Atlantic. We used an average total length of each copepod species after careful consideration of the literature using a procedure similar to Beaugrand *et al*.^43^. We therefore did not measure all individual copepods but used instead an average total length that we then multiplied by the number of individuals. The index of averaged small and large community size was calculated using the average size of each selected taxon^44^. The two size-class indices were considered to be a proxy for mesozooplankton biomass for each sample as follows:

$$\sum _{i=1}^{n}N{b}_{i}{S}_{i}$$

where *n* is the number of different taxa in a given sample, *Nb* is the number of individuals for a given taxa and *S* is the associated average size of the given taxa. Finally, both size-class indices were standardised to their respective 99.9th percentile value to remove the effect of potential outliers and summed to correct for differences in organism size. The sum of both standardised size-class indices represents the mesozooplankton biomass index used in the analysis (Fig. 1). This relative index of biomass therefore has values between mostly 0 and 1, although maximum levels may be slightly above 1 due to the sum of the standardised size-class indices.

To avoid potential bias in biomass sampled between night and day due to diel vertical migration (see supplementary information), only night samples were used in the model training, i.e. when central sampling time is not within 12.00_Local time_ ± (Day Length/2), with Day Length the day’s duration in hours. The full night and day data were used to assess our model’s performance. The model grid cells (of 1/24° resolution) that best represented each CPR sample of 10 nautical miles were identified using the sample’s central position and direction (Fig. SI 3). Environmental data were extracted and averaged over these cells for each sample. Finally, redundancy filtering ensured that observations collected on the same day were separated by more than 2.3 km, i.e. about half the width of a grid cell.

#### Chlorophyll-a data

Daily CHL (mg m^−3^) data were gathered from the MODIS-Aqua (2002–2017; 1/24° resolution) ocean colour sensor using the Ocean Colour Index (OCI) algorithm^45^ and extracted from the NASA portal (https://oceancolour.gsfc.nasa.gov/cgi/l3) with the reprocessing of June 2015. Meso-scale CHL fronts were identified using daily CHL data. These data were pre-processed using iterations of a median filter in order to recover missing data on the edge of the valid data, followed by a Gaussian smoothing procedure to remove eventual sensor stripes^46^. Chlorophyll-a gradient was derived from the daily CHL data using a bi-directional gradient norm over a 3 by 3 grid-cell window as follows:

$$gradCHL=\sqrt{D{x}^{2}+D{y}^{2}}$$

with Dx, Dy, the longitudinal and latitudinal CHL horizontal gradient respectively corrected by the pixel size in km. Small and large chlorophyll-a fronts refer to variable levels of chlorophyll-a gradient (gradCHL).

### Step 2 – Characterisation of mesozooplankton environmental envelope

We considered here the matchups of CHL and gradCHL with the mesozooplankton observations to identify the threshold values that characterised suitable versus unsuitable feeding habitats. Because mesozooplankton biomass was composed of the most frequent 131 species of the North Atlantic, it was assumed that each niche – and its overlap with prey – was covered by at least one species and therefore no physical variables were presently considered as a limiting factor of suitable habitat. However, several variables were shown as illustrative variables in the supplementary information (Fig. SI 4).

The links between the biotic variables (CHL and gradCHL) and positive-only mesozooplankton biomass were analysed using a cluster analysis (the k-means clustering technique; Fig. 2; see supplementary information for full description). The modelling of favourable feeding habitat was primarily driven by the size of chlorophyll-a fronts, i.e. the gradCHL values mostly defined the 0 to 1 daily habitat level. In parallel, the range of chlorophyll-a values was only used to exclude unfavourable habitat such as extremely low productive and potentially eutrophicated areas. We used boundary values from the extreme clusters (i.e. low and high levels) to capture the widest possible environmental envelope of mesozooplankton. Compared with similar habitat studies on HTL species^13,15,17^, we selected more stringent thresholds for gradCHL and CHL (i.e. the 3rd percentile value of the lower and 97th of the upper cluster, instead of the 15th or 20th and 85th ones). The stricter selection was imposed by the low biomass levels that are currently accounted for (instead of presence-only) and the more homogeneous response to productivity fronts of mesozooplankton than mobile top predators. Finally, the 3rd to 97th percentile levels were chosen rather that the common 1st to 99th or 5th to 95th percentile levels as the corresponding thresholds appeared either to be too strict or loose when applied on extreme clusters to correctly capture the environmental envelope. The expert knowledge and response of the observation dataset to these thresholds were preferred over statistical standards to retain the suitable levels. Overall, these thresholds were selected to ensure that extreme suitable environments were represented while rejecting the distribution tails of these extreme clusters. These tails are likely to correspond to outliers from, for example, unusual environments, possible errors in the observation data or misclassified data in the clustering.

### Step 3 – Formulation of the habitat index equation

The mesozooplankton environmental envelope for CHL predicted the daily suitability of each grid cell, assigning a binary habitat value (0 or 1), depending on whether its value is outside (0) or inside (1) the relevant range (Fig. 3b). Concerning the envelope for gradCHL, values were translated into continuous levels between 0 and 1 to account for the various feeding opportunities that exist between the small and large productivity fronts (Fig. 3a). We defined a daily feeding habitat index that represented increasing levels of potential food availability from small to large CHL fronts. The high values of the habitat suitability index revealed large frontal systems that correspond, due to their size and persistence, to water masses supporting the development of abundant mesozooplankton biomasses. Lower values of habitat index refer to smaller, potentially less persistent and productive frontal systems. A daily feeding habitat index was thus defined in each grid cell as satisfying all the suitable environmental conditions, following the equation:

$$Feeding\,Habita{t}_{Day,Cell}=\{\begin{array}{cc}0 & {\rm{i}}{\rm{f}}\,gradCHL < gradCH{L}_{{\min }}\,{\rm{or}}\,CHL\{\,\begin{array}{c} < CH{L}_{{\min }}\\ \, > CH{L}_{{\max }}\end{array}\,\\ 0\,to\,{1}^{\ast } & {\rm{if}}\,gradCH{L}_{{\min }} < gradCHL < gradCH{L}_{{int}}\,{\rm{and}}\,CH{L}_{{\min }} < CHL < CH{L}_{{\max }}\\ 1 & {\rm{if}}\,gradCHL > gradCH{L}_{{int}}\,{\rm{and}}\,CH{L}_{{\min }} < CHL < CH{L}_{{\max }}\end{array}$$

*linear function from 0 to 1 as follows:

$$Feeding\,Habita{t}_{Day,Cell}=1+\frac{\mathrm{ln}(gradCHL)-\,\mathrm{ln}(gradCH{L}_{int})}{\mathrm{ln}(gradCH{L}_{int})-\,\mathrm{ln}(gradCH{L}_{min})}$$

where *ln* is the natural logarithm, *CHL*_*min*_ and *CHL*_*max*_ define the suitable range of chlorophyll-a and *gradCHL*_*min*_ and *gradCHL*_*int*_ are the minimum and intermediate thresholds of CHL gradient that bound the linear function of increasing suitable habitat index between 0 and 1.

Areas meeting the daily requirements of the habitat index were then integrated over time to create seasonal suitability maps of feeding habitat. This time composite is expressed as a frequency of occurrence of daily habitat values, i.e. the sum of the daily habitat values (from 0 to 1) over the number of days for which the habitat suitability index was effectively estimated (Fig. 4).

### Step 4 – Comparison between biomass index and environmental envelope

Model performance was evaluated with abundance data in two ways: (i) comparing gradCHL distribution with the training abundance data (night data, Fig. 3a) and (ii) comparing the 3-day habitat levels with the full data set of the index of mesozooplankton positive biomass (night and day data; Fig. 6). In the first case, the comparison investigated how the daily habitat suitability index, which was derived from gradCHL based on presence data, was linked with abundance data. In the latter case, the performance was tested using a substantially larger number of predicted/observed matchups than for the model training. These matchups were presented as boxplots of biomass by 10th percentile width of habitat index (Fig. 6).

## Data Availability

Data on mesozooplankton habitat available upon reasonable request and monthly composites are available on the JRC data catalogue (https://data.jrc.ec.europa.eu/). Chlorophyll-a data are available online (https://oceancolour.gsfc.nasa.gov/cgi/l3). Mesozooplankton index data are available online (https://www.mba.ac.uk/).

## Supplementary information

Satellite-based indicator of zooplankton distribution for global monitoring. Supplementary information
