# Supplementary material for: Satellite-based indicator of zooplankton distribution for global monitoring
Source: Sci Rep. 2019 Mar 18;9:4732. doi: 10.1038/s41598-019-41212-2 (PMC6427021; doi:10.1038/s41598-019-41212-2)
Supplement: Supplementary file 1 — Satellite-based indicator of zooplankton distribution for global monitoring. Supplementary information [file 41598_2019_41212_MOESM1_ESM.docx]

Satellite-based indicator of zooplankton distribution for global monitoring

Druon J.N., Hélaouët P., Beaugrand G., Fromentin J.M., Palialexis A., Hoepffner N.

# **Supplementary information**

## The Continuous Plankton Recorder

The Continuous Plankton Recorder (CPR) survey is among the few plankton monitoring programmes operated at a basin scale (another example being the California Cooperative Oceanic Fisheries Investigations [Calcofi]). The survey has monitored both phytoplankton and zooplankton monthly since the 1930s using an instrument (the CPR machine) that can be towed at high speed (about 15–20 knots) by merchant ships. This methodology has remained virtually unchanged since 1958, providing one of the most extensive ecological data sets available to the marine ecology community^1–3^. The length of the towing cable is calculated to produce a towing depth of about 10 m^4^ at the operating speed of the vessel. Behind the ship, the upper layer of the water column is mixed due to the turbulent nature of the ship’s wake and it is therefore accepted that the CPR samples the integrated first 10 m of the water column^3^. Water enters through the front 1.27 cm^2^ aperture of the CPR machine and is filtered through a continuously moving band of silk (i.e. the filtering silk). Filtered plankton are subsequently covered by a second moving band of silk (i.e. the covering silk). This sandwich is automatically rolled onto the storage tank containing a dilute solution of borax-buffered formaldehyde (~4%) that fixes the plankton^1^. In the laboratory the silk is cut into samples, corresponding to approximatively 10 nautical miles of sampling (or 18.52 km), before a microscopic procedure is carried out to ensure identification of the zooplankton to the highest practical level.

## Species inserted in the small and large mesozooplankton biomass indices

Two planktonic groups were created for the small (generally below 2 mm; 40 taxa) and large (generally above 2 mm; 91 taxa) mesozooplankton (Table SI 1 and Table SI 2), representing the most present species in the North Atlantic (Fig. SI 2). As mentioned in Richardson et al. 2006^5^, “*Copepods identified usually represent Copepodite Stage V [(pre-adults)] and adults both because the CPR preferentially retains these larger copepods (Robertson, 1968*^6^*) and because they are easier to speciate than juveniles. In addition, females are generally more easily identified to species than are males. For example, females of three smaller species within the genus Pleuromamma are speciated (‘‘Pleuromamma borealis’’, ‘‘Pleuromamma gracilis’’, ‘‘Pleuromamma piseki’’), whereas their males are normally recorded as ‘‘Pleuromamma spp.’’. Males of other genera such as Euchaeta are readily identifiable and are included with the females*”.

Table SI 1: List of the 40 relatively small taxa (generally below 2 mm) present in the whole dataset (n = 68,364) with associated average size (‘CPR traverse’ analysis) and relative contribution (species number multiplied by size in % of all species) for this size class.

| **Taxon** | **Size** | **Contribution** | **Taxon** | **Size** | **Contribution** |
| --- | --- | --- | --- | --- | --- |
|  | **(mm)** | **to index (%)** |  | **(mm)** | **to index (%)** |
| Para*-Pseudocalanus* spp*.* | 1.45 | 27.3666 | *Ctenocalanus vanus* | 1.4 | 0.0320 |
| *Acartia* spp*.* | 1.4 | 19.9730 | *Calocalanus* spp*.* | 1 | 0.0199 |
| *Centropages typicus* | 1.4 | 17.8192 | *Ctenocalanus* spp*.* | 1.25 | 0.0136 |
| *Temora longicornis* | 1.25 | 11.1232 | *Acartia danae* | 1 | 0.0104 |
| *Oithona* spp*.* | 1.15 | 7.2734 | *Euterpina acutifrons* | 0.65 | 0.0097 |
| *Pseudocalanus* spp*.* | 1.45 | 6.0711 | *Clytemnestridae* | 1 | 0.0064 |
| *Clausocalanus* spp*.* | 1.25 | 3.3502 | *Tortanus discaudatus* | 2.25 | 0.0054 |
| *Centropages hamatus* | 1.15 | 1.5017 | *Acartia longiremis* | 0.95 | 0.0050 |
| *Paracalanus* spp*.* | 1 | 1.4137 | *Lubbockia* spp*.* | 2 | 0.0032 |
| *Centropages* spp*. (Unidentified)* | 1.6 | 1.0875 | *Macrosetella gracilis* | 1.35 | 0.0022 |
| *Corycaeus* spp*.* | 1.8 | 0.9310 | *Microcalanus* spp*.* | 0.85 | 0.0020 |
| *Oncaea* spp*.* | 0.95 | 0.7835 | *Parapontella brevicornis* | 1.45 | 0.0019 |
| *Lucicutia* spp*.* | 5.65 | 0.3844 | *Temora turbinata* | 1.45 | 0.0015 |
| *Microsetella* spp*.* | 0.55 | 0.1906 | *Acartia negligens* | 1.45 | 0.0015 |
| *Isias clavipes* | 1.5 | 0.1655 | *Hemicyclops aberdonensis* | 1.45 | 0.0015 |
| *Scolecithricella* spp*.* | 3.35 | 0.1638 | *Pseudocalanus* adult Pacific | 1.45 | 0.0008 |
| *Temora stylifera* | 1.65 | 0.1228 | *Diaixis hibernica* | 0.95 | 0.0005 |
| *Centropages chierchiae traverse* | 1.9 | 0.1066 | *Acartia* spp. – Antarctic | 1.4 | 0.0004 |
| *Mecynocera clausi* | 1.05 | 0.0533 | *Pseudodiaptomus* spp*.* | 1.3 | 0.0003 |
| *Ctenocalanus vanus* | 1.4 | 0.0320 | *Acartia tonsa* | 1.15 | 0.0003 |
| *Calocalanus* spp*.* | 1 | 0.0199 | *Farranula* spp*.* | 0.85 | 0.0002 |

Table SI 2: List of the 91 relatively large taxa (generally above 2 mm) present in the whole dataset (n = 68,364) with associated average size (‘CPR eye-count’ analysis) and relative contribution (species number multiplied by size in % of all species) for this size class.

| **Taxon** | **Size** | **Contribution** | **Taxon** | **Size** | **Contribution** |
| --- | --- | --- | --- | --- | --- |
|  | **(mm)** | **to index (%)** |  | **(mm)** | **to index (%)** |
| *Calanus finmarchicus* | 3.7 | 65.09250 | *Candacia pachydactyla* | 2.45 | 0.00853 |
| *Calanus helgolandicus* | 2.7 | 17.33127 | *Candacia bipinnata* | 2.55 | 0.00845 |
| *Metridia lucens* | 2.75 | 5.35962 | *Alteutha* spp*.* | 0.9 | 0.00799 |
| *Paraeuchaeta norvegica* | 8.25 | 3.84413 | *Euchaeta media* | 3.95 | 0.00716 |
| *Calanus hyperboreus* | 7.65 | 1.01623 | *Eucalanus hyalinus* | 5.75 | 0.00686 |
| *Euchaetidae* | 6.85 | 0.96420 | *Miracia efferata* | 1.7 | 0.00677 |
| *Pleuromamma borealis* | 1.95 | 0.89215 | *Centropages violaceus* | 2 | 0.00639 |
| *Paraeuchaeta hebes* | 3.15 | 0.70059 | *Rhincalanus cornutus* | 3.3 | 0.00630 |
| *Pleuromamma gracilis* | 2.05 | 0.51751 | *Aetideus armatus* | 1.8 | 0.00528 |
| *Nannocalanus minor* | 1.85 | 0.45241 | *Undeuchaeta* spp*.* | 4.75 | 0.00499 |
| *Candacia armata* | 2.45 | 0.41125 | *Paracandacia bispinosa* | 1.8 | 0.00438 |
| *Calanus glacialis* | 4.55 | 0.35729 | *Undeuchaeta major* | 4.75 | 0.00431 |
| *Pleuromamma abdominalis* | 3.45 | 0.32992 | *Labidocera* spp*.* | 2.9 | 0.00374 |
| *Metridia longa* | 3.05 | 0.32463 | *Heterorhabdus* spp*.* | 3.1 | 0.00370 |
| *Pleuromamma robusta* | 3.4 | 0.31022 | *Subeucalanus monachus* | 2.3 | 0.00335 |
| *Pleuromamma piseki* | 2.05 | 0.27878 | *Paraeuchaeta* spp*.* | 6.8 | 0.00276 |
| *Neocalanus gracilis* | 3 | 0.20123 | *Parathalestris croni* | 2 | 0.00229 |
| *Euchaeta acuta* | 4 | 0.18701 | *Candacia curta* | 2.2 | 0.00184 |
| *Euchirella rostrata* | 3.05 | 0.15824 | *Paracandacia* spp*.* | 1.85 | 0.00137 |
| *Calanoides carinatus* | 2.8 | 0.15021 | *Pseudodiaptomus* spp*.* | 1.3 | 0.00130 |
| *Centropages chierchiae* | 1.9 | 0.14811 | *Neocalanus robustior* | 3.75 | 0.00125 |
| *Pleuromamma xiphias* | 4.85 | 0.09949 | *Aetideus giesbrechti* | 1.65 | 0.00102 |
| *Undeuchaeta plumosa* | 3.75 | 0.09527 | *Paraeuchaeta gracilis* | 6.05 | 0.00101 |
| *Euchaeta marina* | 3.1 | 0.08600 | *Scolecithrix* spp*.* | 3.5 | 0.00092 |
| *Sapphirina* spp*.* | 4.5 | 0.07997 | *Pontellina plumata* | 1.45 | 0.00083 |
| *Rhincalanus nasutus* | 4.4 | 0.07662 | *Euchirella messinensis* | 4.5 | 0.00075 |
| *Candacia* spp*.* | 3.15 | 0.07048 | *Candacia longimana* | 3.15 | 0.00075 |
| *Candacia ethiopica* | 2.5 | 0.04091 | *Scaphocalanus echinatus* | 1.95 | 0.00074 |
| *Undinula vulgaris* | 2.55 | 0.03917 | *Monstrilloida* | 3.25 | 0.00070 |
| *Scolecithrix danae* | 2.1 | 0.03376 | *Scolecithrix bradyi* | 1.3 | 0.00065 |
| *Subeucalanus crassus* | 3.5 | 0.03056 | *Subeucalanus pileatus* | 2.1 | 0.00040 |
| *Centropages bradyi* | 1.9 | 0.02837 | *Scaphocalanus* spp*.* | 3.35 | 0.00040 |
| *Mesocalanus tenuicornis* | 2.45 | 0.02279 | *Haloptilus spiniceps* | 4.05 | 0.00029 |
| *Metridia* spp. (V-VI) | 5.9 | 0.02167 | *Subeucalanus mucronatus* | 3 | 0.00029 |
| *Labidocera wollastoni* | 2.3 | 0.01860 | *Heterorhabdus oikoumenikis* | 2.8 | 0.00027 |
| *Anomalocera patersoni* | 3.3 | 0.01551 | *Aetideus* spp*.* | 2.15 | 0.00026 |
| *Euchirella* spp*.* | 5.35 | 0.01289 | *Aetideus acutus* | 1.6 | 0.00011 |
| *Labidocera aestiva* | 2.4 | 0.01265 | *Augaptilus* spp*.* | 4.25 | 0.00010 |
| *Corycaeus speciosus* | 1.55 | 0.01191 | *Haloptilus acutifrons* | 3.4 | 0.00008 |
| *Urocorycaeus* spp*.* | 2.15 | 0.01174 | *Euchirella amoena* | 3.35 | 0.00008 |
| *Heterorhabdus norvegicus* | 3.6 | 0.00953 | *Heterostylites longicornis* | 3.3 | 0.00008 |
| *Paracandacia simplex* | 1.95 | 0.00944 | *Pontellopsis regalis* | 3.05 | 0.00007 |
| *Neocalanus* spp*.* | 6 | 0.00930 | *Spinocalanus* spp*.* | 2.95 | 0.00007 |
| *Heterorhabdus papilliger* | 2.15 | 0.00923 | *Heterorhabdus abyssalis* | 2.85 | 0.00007 |
| *Labidocera acutifrons* | 4 | 0.00897 | *Haloptilus longicornis* | 1.9 | 0.00005 |
| *Copilia* spp*.* | 5.2 | 0.00868 |  |  |  |

## Distribution of the mesozooplankton presence data (2002–2016)

**(a)**

**
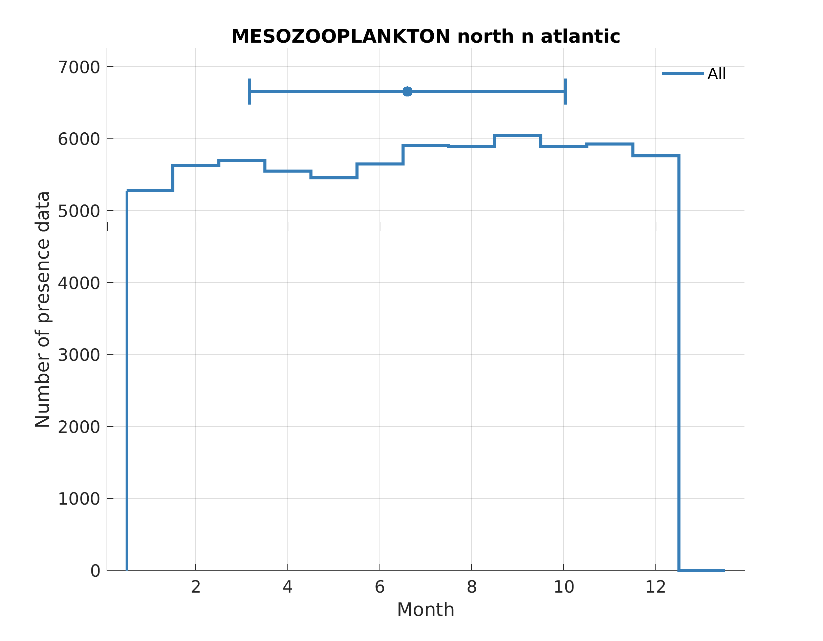

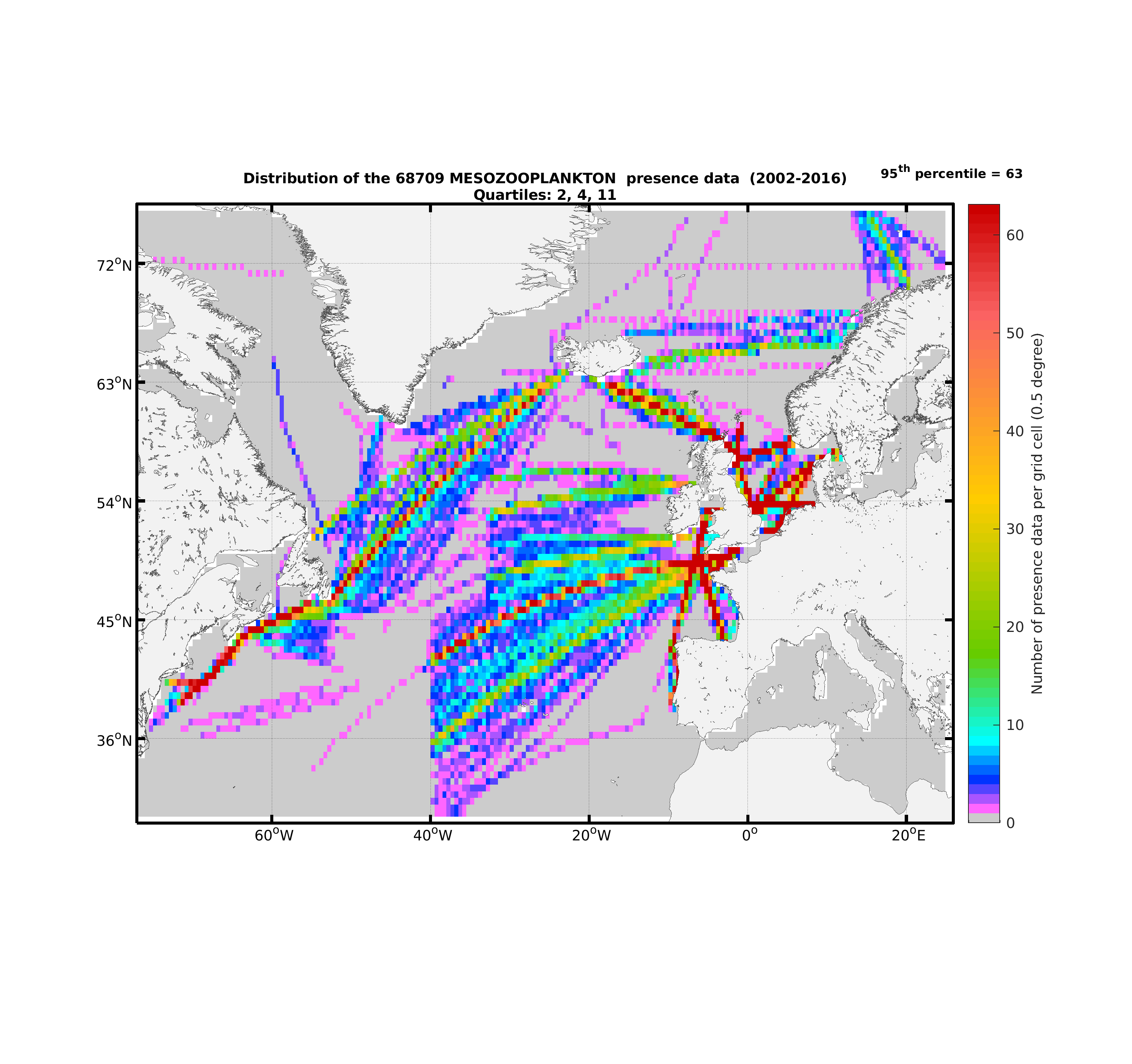
**

**(b)**

Figure SI 1 (a) Monthly and (b) spatial distributions of the CPR sampling from 2002 to 2016 (including absence data, n = 68,709). Only positive abundance data (n = 54,282) were used in the analysis.

## Original mesozooplankton biomass index by size class

**(a)**

**
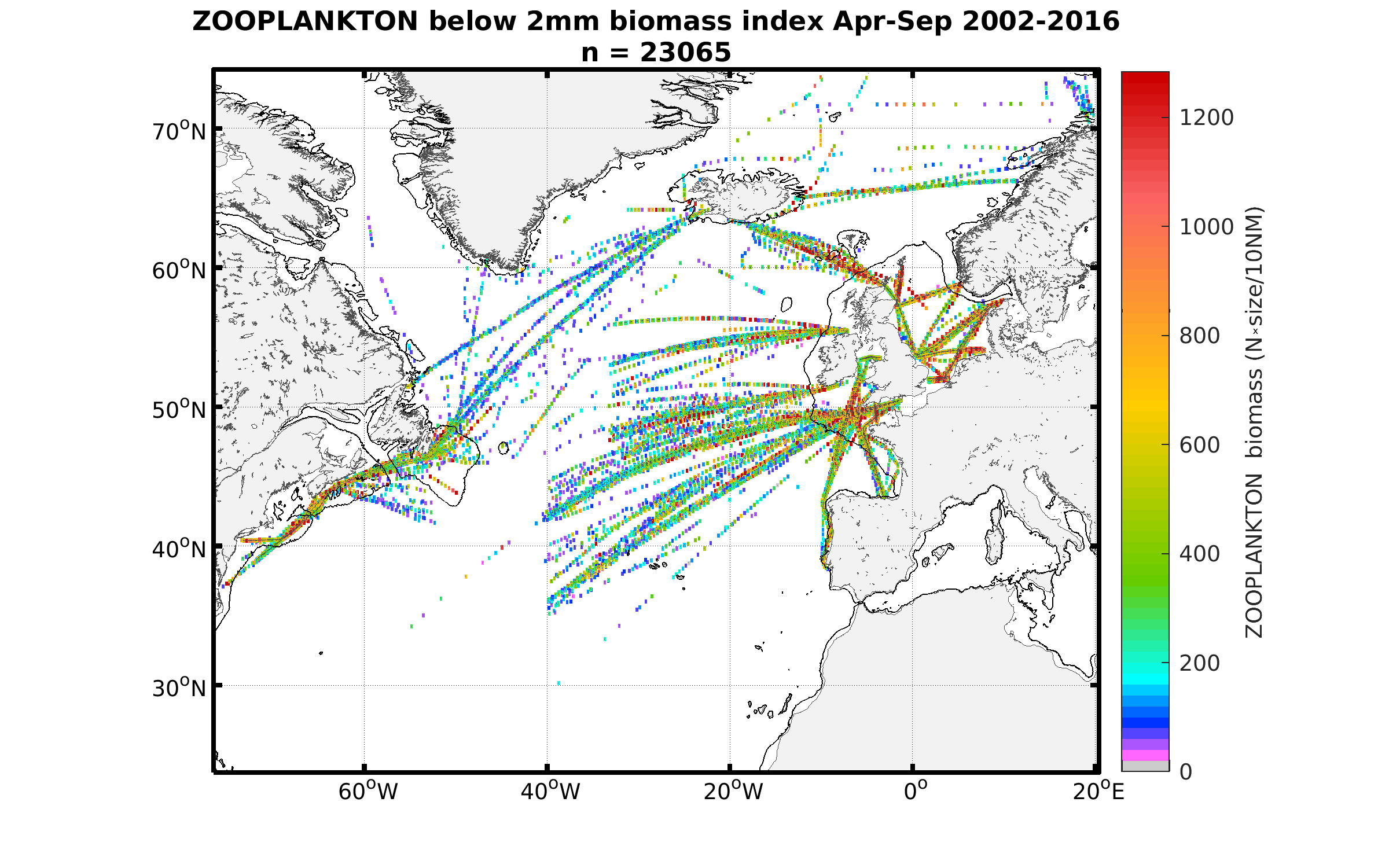
**

**(b)**

**
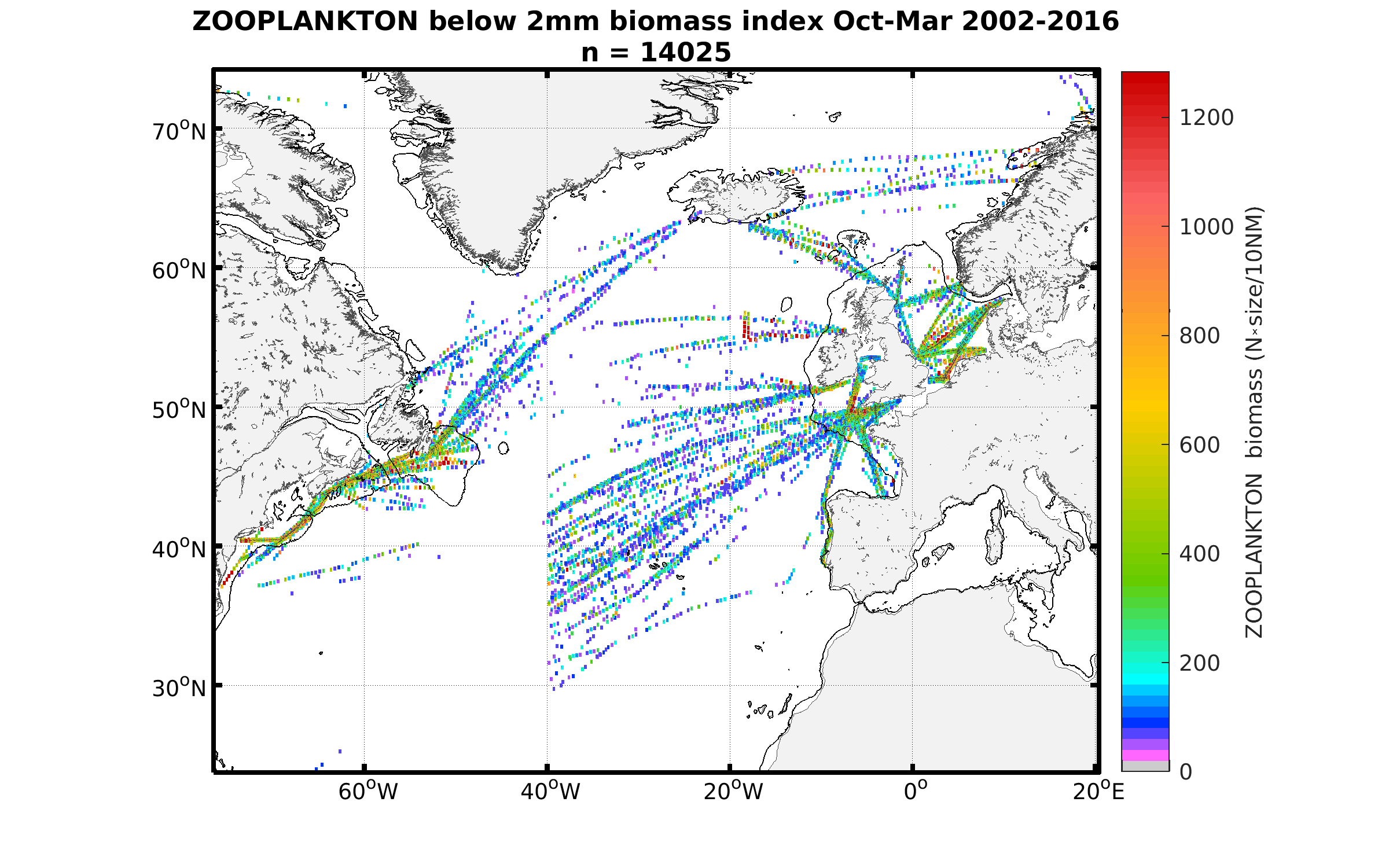
**

**
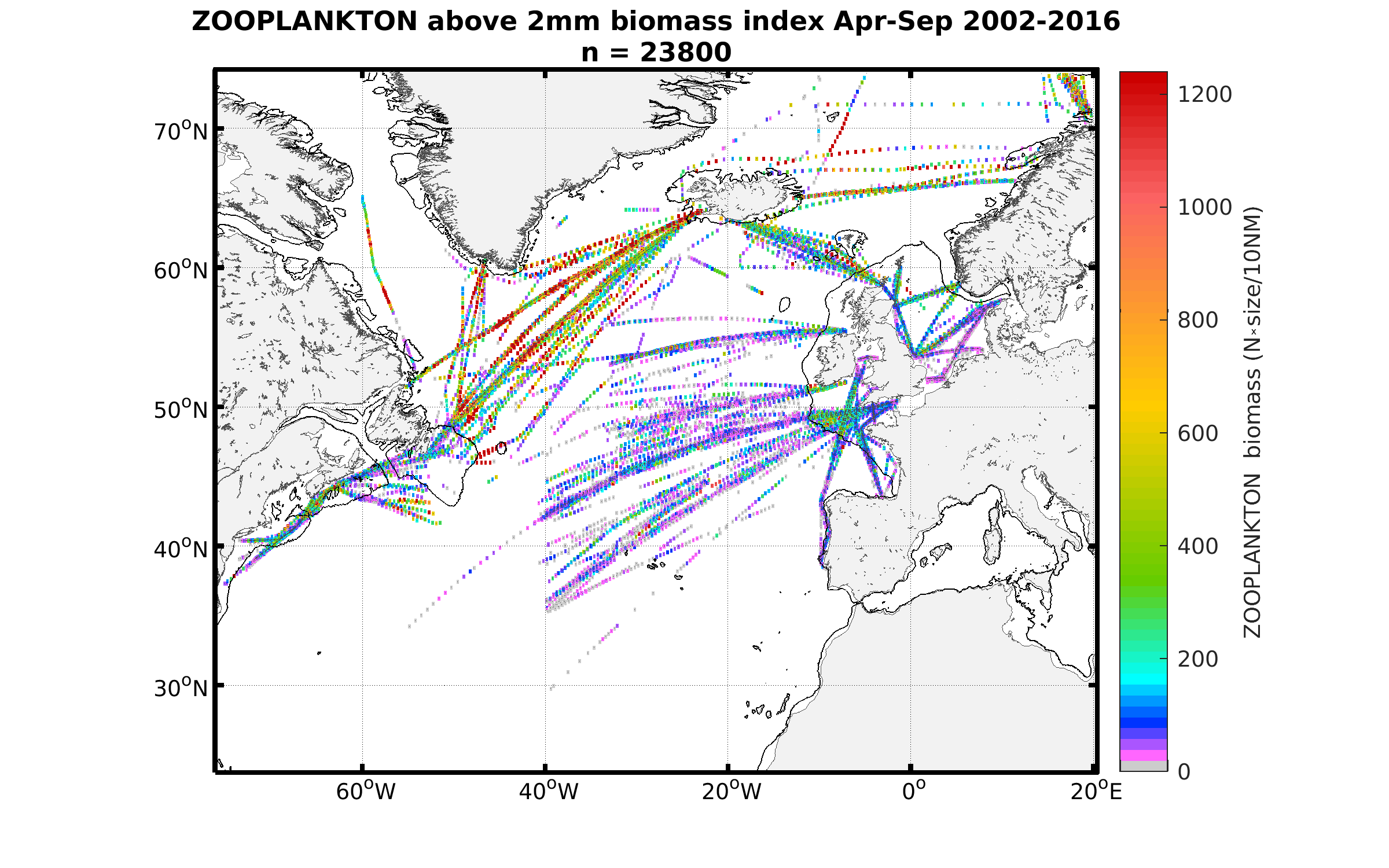
**

**(d)**

**(c)**

**
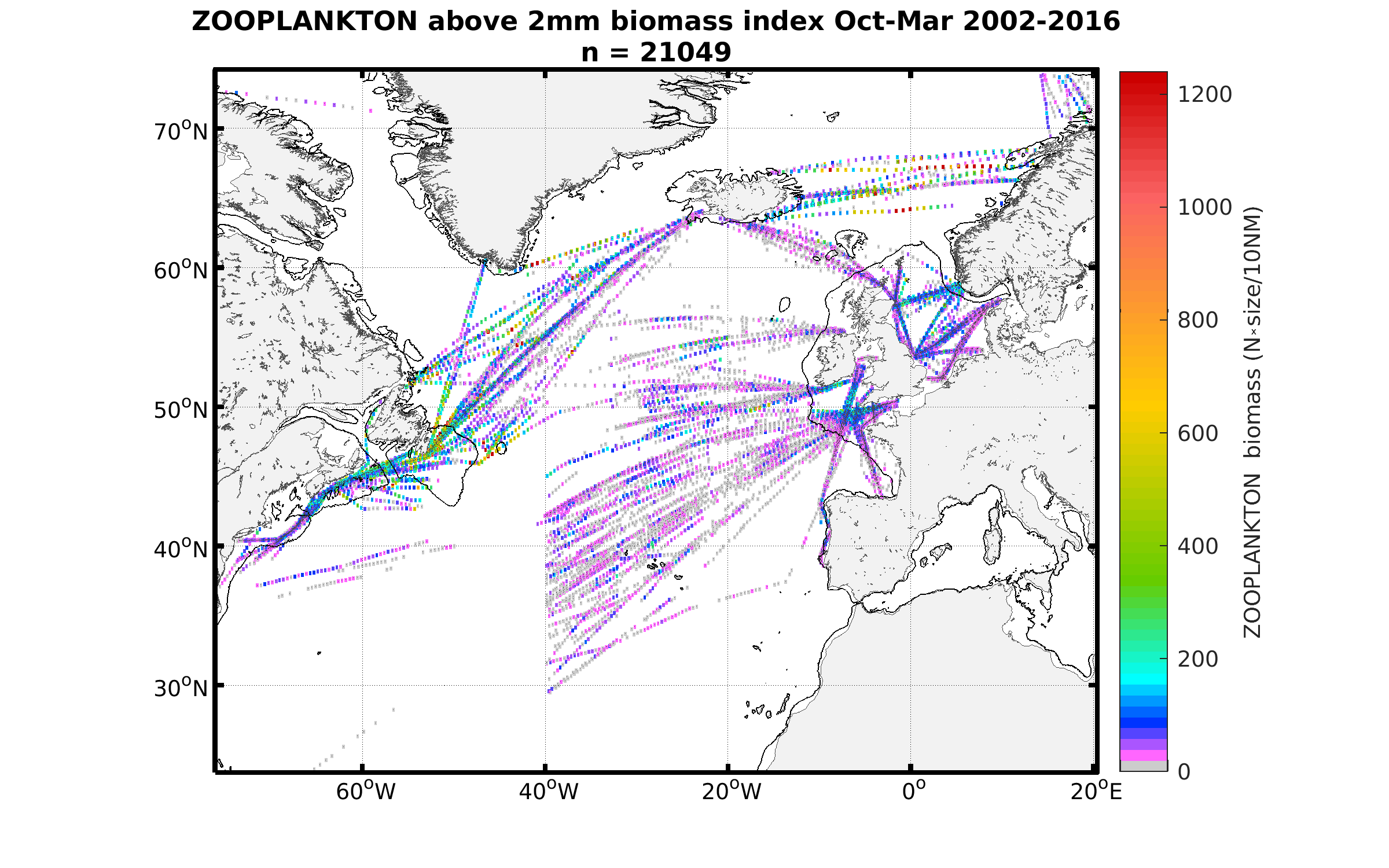
**

Figure SI 2 Geographical mean index of mesozooplankton biomass (positive values only) in the North Atlantic (a, c) for the high (April-September, upper map; n = 23,800) and (b, d) the low (October-March, lower map; n = 21,049) seasons and the (a, b) small and (c, d) large mesozooplankton observations (generally below and above 2 mm size) collected between 2002 and 2016 by the Continuous Plankton Recorder survey (mean relative biomass in cells of 0.3˚ by 0.3˚). The biomass index is expressed in number per mean size and 10 nautical miles’ sample. The maximum value of the colour bar was set to the 95th percentile of the high season (1,225 and 1,230 for the small and large mesozooplankton, respectively).

## Diel vertical migration of mesozooplankton

Diel vertical migration (DVM) is an almost universal feature of zooplankton communities, especially in areas south of the oceanic Polar Front^7^. The main observed DVM pattern is an increase in zooplankton abundance/biomass in the subsurface of the water column during the night followed by a diminution at daylight. The pattern is commonly explained to reduce the risk of mortality by predation. The principal pieces of evidence supporting this hypothesis are (i) the normal DVM is more pronounced for heavily pigmented taxa that are more easily perceived by visual predators^8^ and (ii) migrating zooplankton tends to be near the surface only when illumination levels are low^8^.

## Spatial selection of biomass and environmental data matchups

**(a)
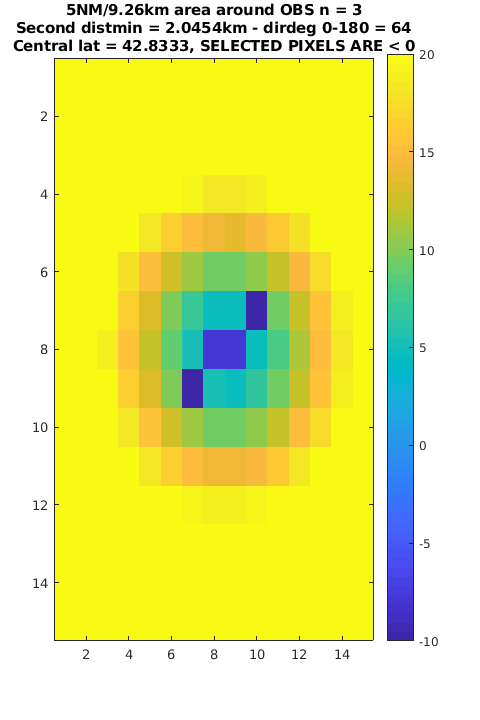
(b)
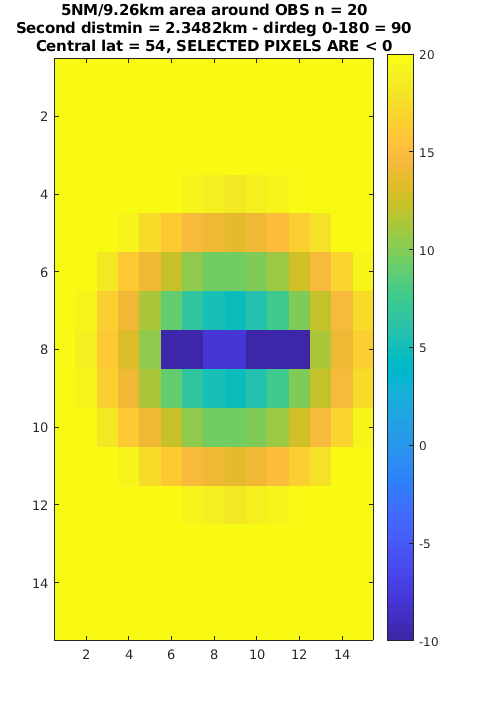
 (c)
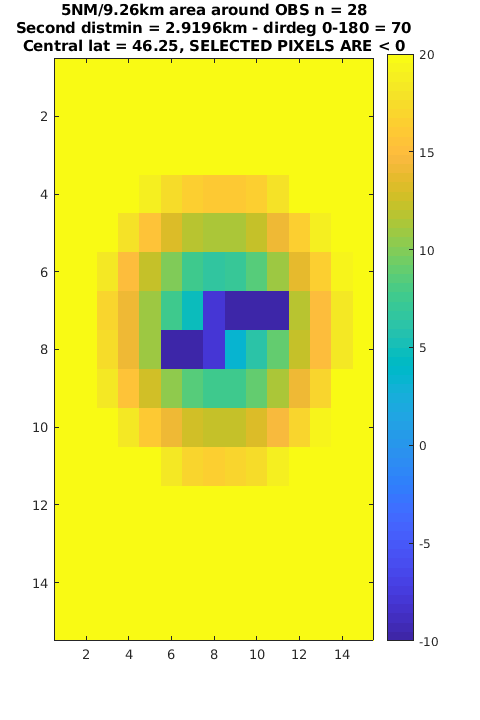
**

Figure SI 3 Examples of matchups (cells in dark blue) between samples of mesozooplankton biomass from the Continuous Plankton Recorder survey of 10 nautical miles length of direction (a) 64, (b) 90 and (c) 70 degrees (central position centred in the central cell of graphs – in position 8, 8) and the selected grid cells of the habitat index (1/24˚ resolution).

## Cluster analysis method and illustrative abiotic variables

Cluster analysis is a suitable method for identifying homogenous groups of objects or ‘clusters’ (here, mesozooplankton habitat suitability), regardless of their respective number. A cluster analysis is well suited to identify habitats that are marginally represented and may otherwise be interpreted as outliers by other statistical methods. The number of clusters was chosen as the minimum number that favours interpretation. We used 3-day mean values of CHL and gradCHL to increase the number of matchups assuming that variability is low at ±1 day from the sampling date and at a spatial resolution of 1/24˚. CHL and gradCHL datasets were log-transformed prior to use, due to the wide variability in their values, and were normalised by the mean and standard deviation prior to performing the cluster analysis.

K-means clustering^9^ based on a Euclidean distance was used to estimate the association of data points between clusters and to minimise the within-cluster sum of squared errors. In k-means clustering, the number of clusters k was first chosen (here the minimum number that favours interpretation) and the cluster centres were initialised randomly. Each data point was then assigned to the closest cluster based on a selected distance measure (similarity) and updated cluster centre. At each iteration step, the new cluster centres were computed as the mean vectors of the assigned data points. These two steps, data point assignment and cluster centre update, were repeated until the cluster centres did not change any more or until a sufficient number of iterations were performed. Matlab’s k-means function was used with 500 iterations/restarts and the Euclidian distance setting. The z-score transformation^10^ was performed before clustering, in which each data variable was normalised to zero mean and unit variance to guarantee that each selected variable had equal influence on the minimisation of the within-cluster sum of squares objective function^10^.

The cluster analysis (Fig. 2) was applied with non-influential (illustrative) variables to reveal the coherence of the results on mesozooplankton habitat suitability with respect to the abiotic conditions. Abiotic variables were extracted from the EU Copernicus Marine Environment Monitoring Service (see description below). The cumulative distribution of illustrative abiotic variables (Fig. SI 4) highlights that the red cluster, corresponding to the lowest values of the index of mesozooplankton biomass, tends to occur mostly in surface waters characterised by warmer temperature, being more deeply-stratified, in further southern areas, with high levels of sea surface height anomaly (SSHa) and salinity (high evaporation), and low oxygen levels compared with the other clusters. These traits indeed characterise relatively poorly productive areas. On the contrary, the cluster of the highest mesozooplankton biomass index (black) tends to occur in generally recently stratified waters of higher latitude, in lower SSHa and temperature levels and longer day length and higher oxygen levels, which are typical traits of productive waters. The green and blue clusters (medium level of mesozooplankton biomass) generally correspond to intermediate levels of abiotic conditions except for salinity and mixed layer depth where the green cluster shows the lowest levels likely in relation to high precipitation and recently formed mixed layer depth (spring bloom).

The comparison of the relative influence of the photosynthetically active radiation (PAR) at sea surface with the day length on each cluster reveals that day length discriminates the high mesozooplankton biomass cluster (black) better. This is probably because surface phytoplankton productivity is generally not limited by the solar radiation near the sea surface, while day length is likely to be a major limitation of primary productivity. Finally, all clusters show a relatively regular sampling from year to year.

Data for the abiotic variables (i.e. SSHa (m), SST (˚C), SSS (psu), MLD (m) and O_2_ (mmol m^–3^), see figure legend of Fig. SI 4) are extracted from the global ocean model (1997–2014) provided by the EU Copernicus Marine Environment Monitoring Service (http://marine.copernicus.eu/). Monthly mean data are extracted from the global model (Glorys2V3) with a 1/4˚ horizontal resolution and 75 unevenly spaced vertical levels. This ocean model includes a variational data assimilation scheme for vertical temperature and salinity profiles and satellite sea level anomalies. These monthly data are interpolated onto the MODIS-Aqua grid (1/24˚ resolution) and then linearly interpolated to obtain daily values that match the sampling day. This month-to-day interpolation step is assumed to produce suitable estimates of the seasonal changes that define habitat. SST and SSS values are calculated from the upper model layer (ca. 3 m) and are thus considered to be representative of the mixed layer. MLD is defined as the maximum of the vertical density gradient. PAR data from the MODIS-Aqua sensor were extracted from the NASA portal (https://oceancolour.gsfc.nasa.gov/cgi/l3).


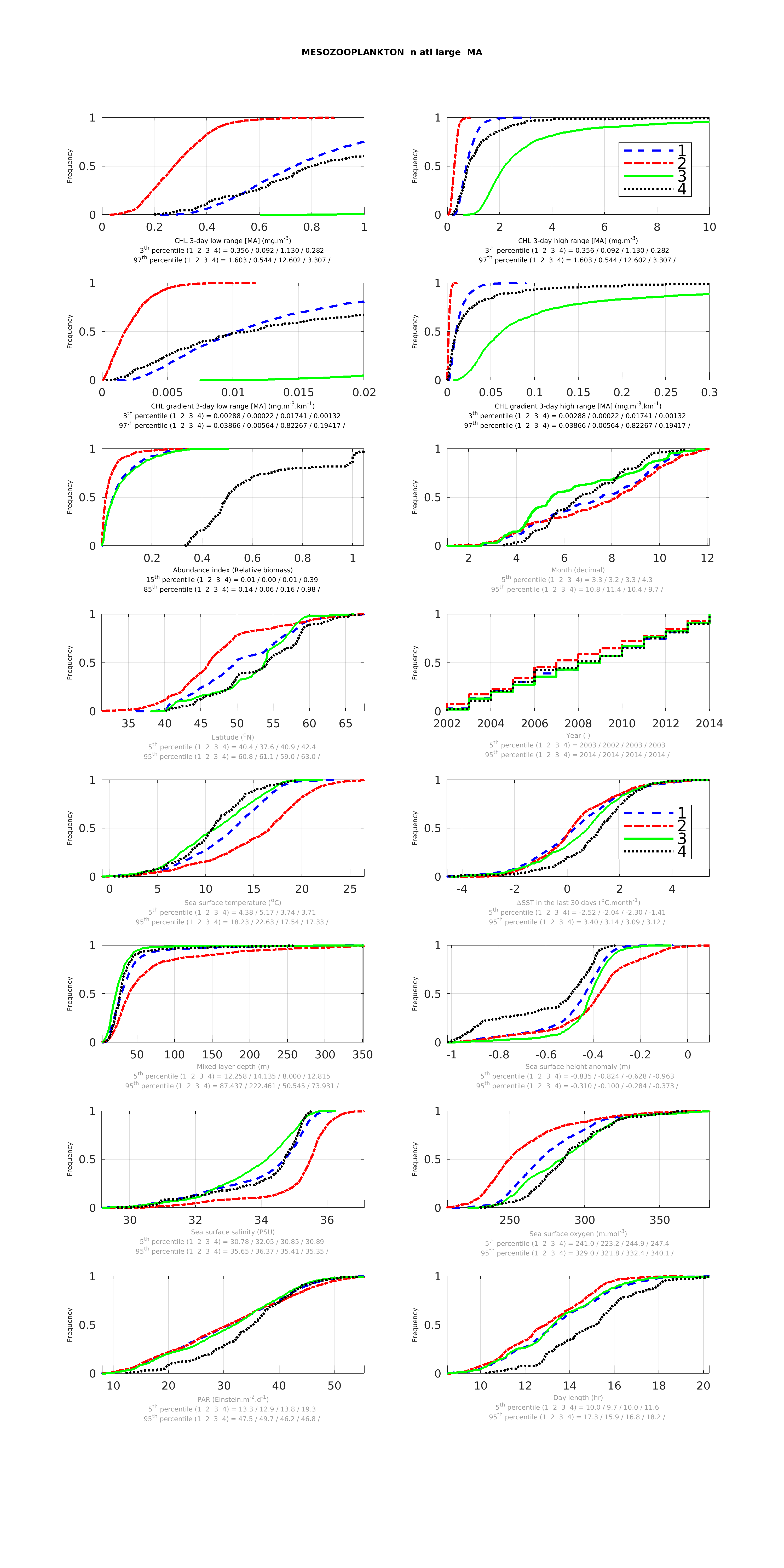


Figure SI 4 Cumulative distributions for the illustrative abiotic variables as output of the main cluster analysis (Fig. 2 of the main publication for details). The illustrative variables are: latitude, year, sea surface temperature (SST), SST increase over the last 30 days (ΔSST), mixed layer depth (MLD), sea surface height anomaly (SSHa), sea surface salinity (SSS), sea surface oxygen (O_2_), photosynthetically active radiation (PAR) and day length.


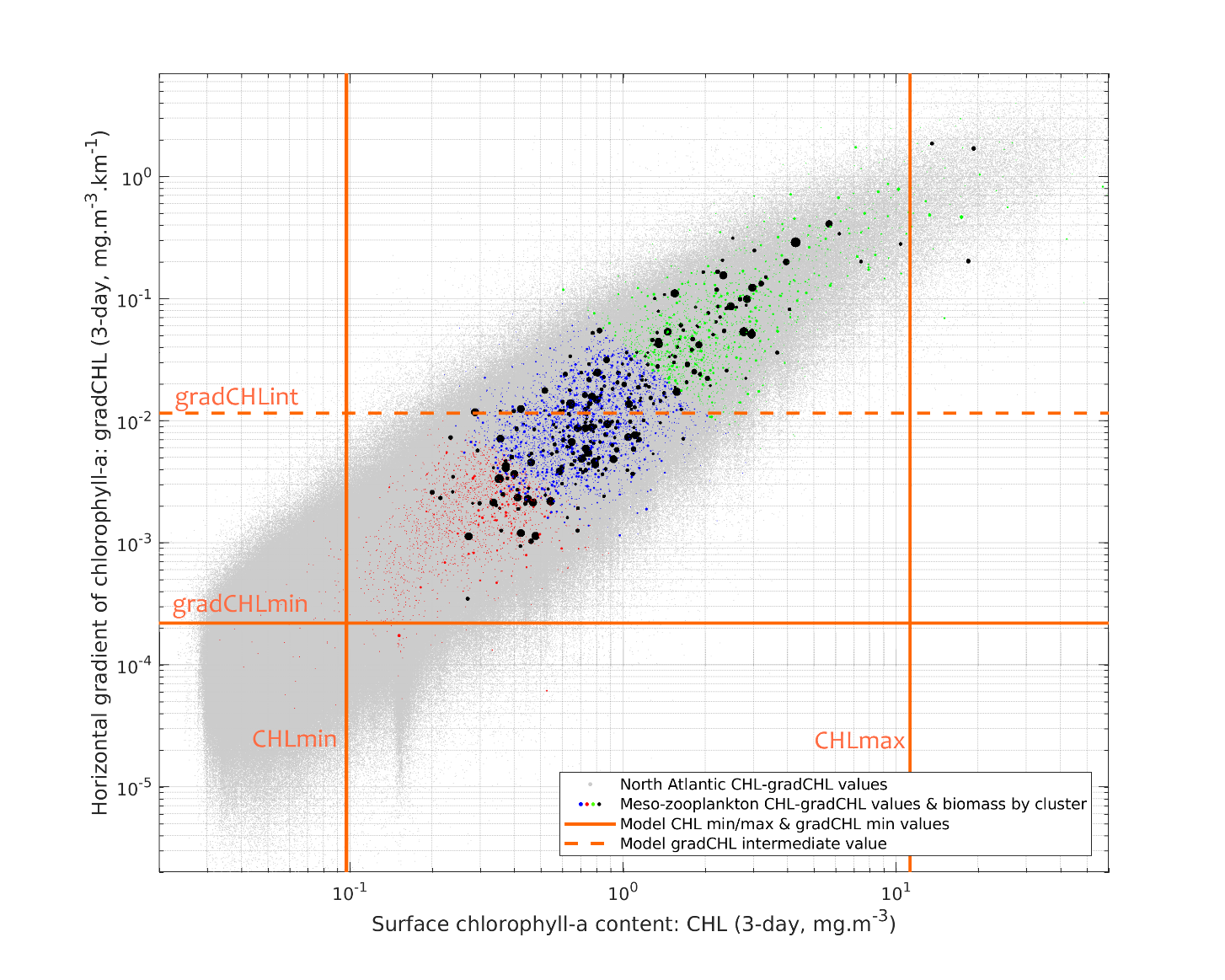


Figure SI 5 Scatter plot (logarithmic scale) of chlorophyll-a content (CHL) versus horizontal gradient of chlorophyll-a (gradCHL) for the entire North Atlantic (grey dots). Mesozooplankton biomass index is superimposed (the size of the dots being proportional to mesozooplankton biomass) with colours according to the clustering (see Fig. 2). The suitable range of CHL, the minimum value of gradCHL (orange solid lines) and the intermediate value of gradCHL (orange dashed line) are superimposed (see Fig. 3).

## Seasonal variability of mesozooplankton habitat


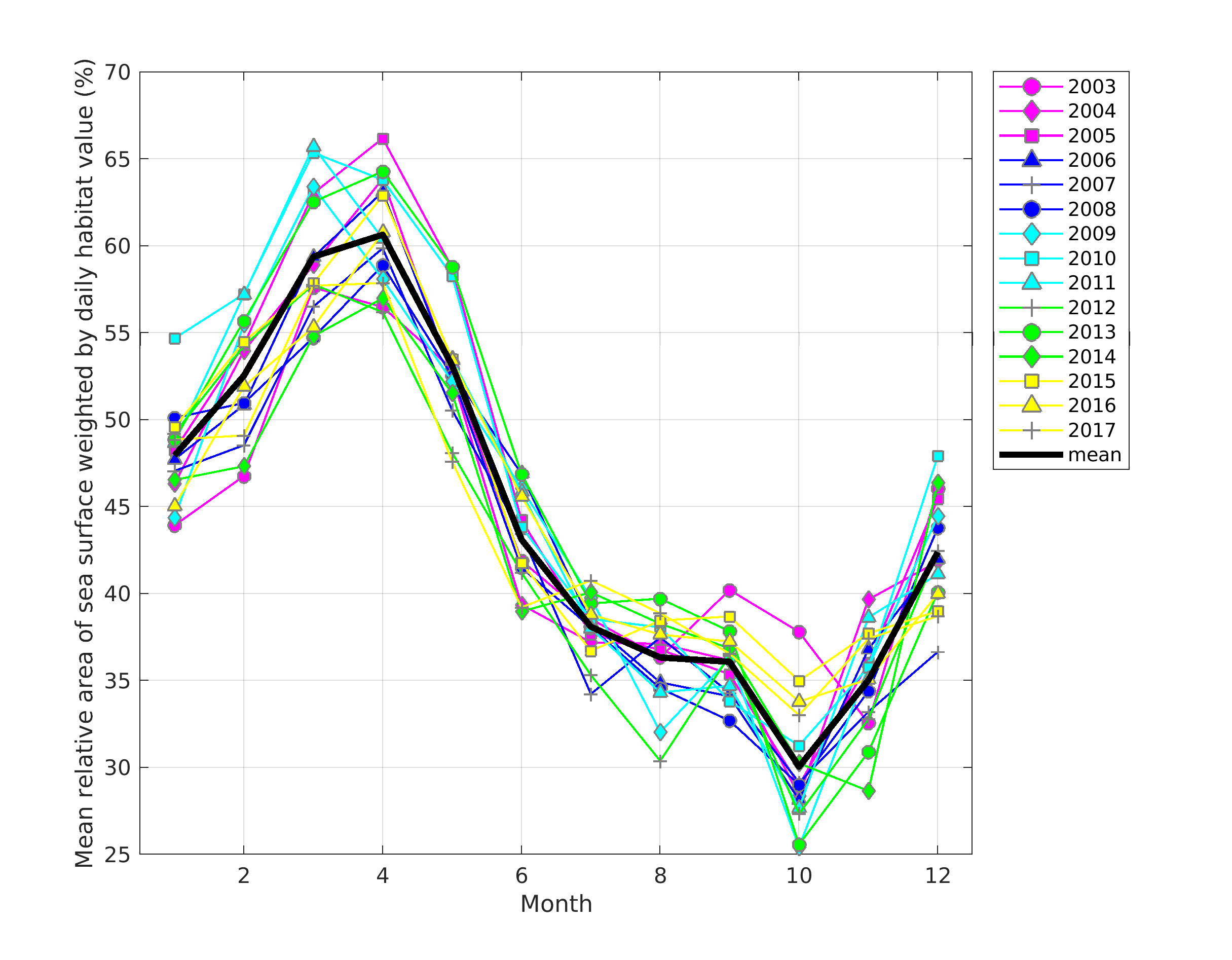


Figure SI 6 Monthly habitat size of mesozooplankton in the North Atlantic Ocean for the period 2003–2017 (values relative to the surface ocean area). The mean seasonal habitat is shown (black solid line).

## References

1. Batten, S. D. *et al.* CPR sampling: the technical background, materials and methods, consistency and comparability. *Prog. Oceanogr.* **58**, 193–215 (2003).

2. Reid, P. C., Colebrook, J. M., Matthews, J. B. L. & Aiken, J. The Continuous Plankton Recorder: concepts and history, from plankton indicator to undulating recorders. *Prog. Oceanogr.* **58**, 117–173 (2003).

3. Warner, A. J. & Hays, G. C. Sampling by the continuous plankton recorder survey. *Prog. Oceanogr.* **34**, 237–256 (1994).

4. Hélaouët, P., Beaugrand, G. & Reygondeau, G. Reliability of spatial and temporal patterns of C. finmarchicus inferred from the CPR survey. *J. Mar. Syst.* **153**, 18–24 (2016).

5. Richardson, A. J. *et al.* Using continuous plankton recorder data. *Prog. Oceanogr.* **68**, 27–74 (2006).

6. Robertson, A. continuous plankton recorder: a method for studying the biomass of calanoid copepods. *Bull. Mar. Ecol.* (1968).

7. Beaugrand, G., Ibañez, F. & Lindley, J. A. Geographical distribution and seasonal and diel changes in the diversity of calanoid copepods in the North Atlantic and North Sea. *Mar. Ecol. Prog. Ser.* **219**, 189–203 (2001).

8. Hays, G. C., Proctor, C. A., John, A. W. G. & Warner, A. J. Interspecific differences in the diel vertical migration of marine copepods: the implications of size, color, and morphology. *Limnol. Oceanogr.* **39**, 1621–1629 (1994).

9. MacQueen, J. Some methods for classification and analysis of multivariate observations. in *Proceedings of the fifth Berkeley symposium on mathematical statistics and probability* **1**, 14 (1967).

10.Berthold, M., Borgelt, C. & Höppner, F. *Guide to intelligent data analysis*. **42**, (Springer, pp. 394, 2010).
